# Pd-Catalyzed Rearrangement Reaction of *N*-Tosylhydrazones Bearing Allyl Ethers Into *Trans*-Olefin-Substituted Sulfonylhydrazones

**DOI:** 10.3389/fchem.2021.782641

**Published:** 2021-10-25

**Authors:** Yaoyao Chang, Jianfang Fu, Yingxue Li, Rongcai Ding, Yue Liu, Jinxing Hu

**Affiliations:** Weifang Medical University, Weifang, China

**Keywords:** *trans*-structure, allyl ethers, sulfonylhydrazones, rearrangement, palladium

## Abstract

A novel and efficient rearrangement of *N*-tosylhydrazones bearing allyl ethers into *trans*-olefin-substituted sulfonylhydrazones is proposed. The reaction involves breakage of the C-O bond and formation of the C-N bond. The reaction can be extended to a wide range of substrates, and the target products can be synthesized smoothly, regardless of the presence of electron-donating and electron-withdrawing groups. The proposed strategy is a new direction in the field of rearrangement reactions.

## Introduction

Hydrazones are a class of Schiff bases with a special molecular structure containing a substructure (-NHN = C-). Many studies have shown that hydrazones possess a wide range of physiological activities, including antioxidant, anti-inflammatory, antibacterial, insecticidal, antiviral, and antitumor activities. In recent years, hydrazones have been highly valued in the fields of medicine, pesticides, materials science, and testing reagents, and have broad development prospects (Yang et al., 1996; Khan, 2008; Özbek et al., 2009; Özdemir et al., 2009; Belkheiri et al., 2010; Özdemir et al., 2010; Özkay et al., 2010).

**SCHEME 1:**
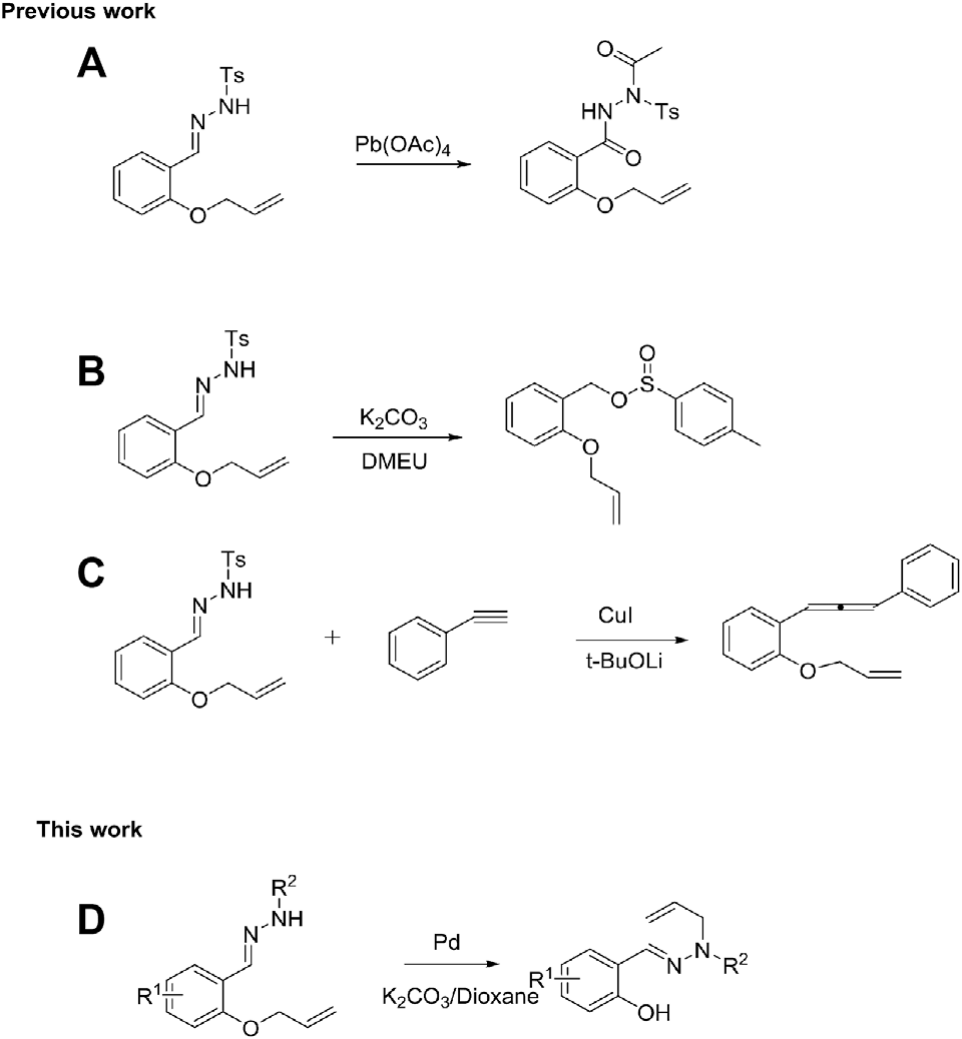
Reaction types with *N*-tosylhydrazones as substrate.

QuinShimizu’s group developed a method for the oxidation of *N*-sulfonyl hydrazide catalyzed by lead tetraacetate (Scheme 1A) (Shimizu et al., 1980). Subsequently, Ashok et al. established a new scheme for the synthesis of sulfinates (Scheme 1B) through the K_2_CO_3_-catalyzed rapid conversion of *N*-sulfonyl hydrazide (Korawat and Basak, 2020). Hossain et al. reported a synthetic route to 1,3-disubstituted allenes through the CuI-catalyzed cross-coupling of *N*-tosylhydrazones with terminal alkynes (Scheme 1C) (Hossain et al., 2013). Furthermore, palladium-catalyzed allylation is a reliable and widely used method (Trost et al., 2006; Lu and Ma, 2007; Mohr and Stoltz, 2007; Weaver et al., 2011)and has been extensively used in total synthesis (Trost and Crawley, 2003; Enquist and Stoltz, 2008; Huters et al., 2012). Therefore, metal-catalyzed cleavage of C-O bonds of ethers remains an intriguing topic. Herein, we report the Pd-catalyzed rearrangement of *N*-tosylhydrazones bearing allyl ethers to produce *trans*-olefin-substituted sulfonylhydrazones (Scheme 1D).

## Results and Discussion

When the reaction was carried out with *N*-tosylhydrazones **1a** in the presence of Pd(PPh_3_)_4_ in THF and K_2_CO_3_ as the base, the desired product (*E*)-*N*-allyl-*N'*-(2-hydroxybenzylidene)-4-methylbenzene sulfonohydrazide **2a** was obtained. Different catalysts were screened for the reaction, such as Pd(OAc)_2_, Pd(PPh_3_)Cl_2_, PdCl_2_, Pd_2_ (dba)_3_, and Pd(PPh_3_)_4_. Among these, Pd(PPh_3_)_4_ proved to be the best catalyst, which led to 55% yield of the final compound (Table 1, entries 2–6). When the reaction was carried out in the absence of a catalyst, the target compound was not obtained (Table 1, entry 7).

**TABLE 1:** Screening of reaction conditions^a^.
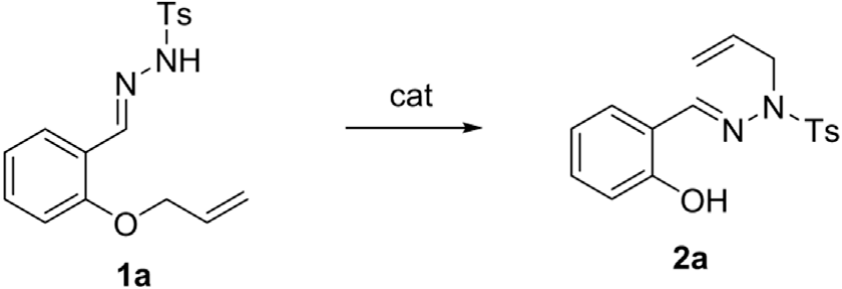

Entry	Catalysts	Additives	Solvent	Yield (%)^b^
1	Pd(PPh_3_)_4_	K_2_CO_3_	THF	55
2	Pd(OAc)_2_	K_2_CO_3_	THF	0
4	Pd(PPh_3_)Cl_2_	K_2_CO_3_	THF	0
5	PdCl_2_	K_2_CO_3_	THF	45
6	Pd_2_ (dba)_3_	K_2_CO_3_	THF	42
7	-	K_2_CO_3_	THF	0
8	Pd(PPh_3_)_4_	K_2_CO_3_	EtOAc	26
9	Pd(PPh_3_)_4_	K_2_CO_3_	Dioxane	65
10	Pd(PPh_3_)_4_	K_2_CO_3_	Toluene	41
11	Pd(PPh_3_)_4_	K_2_CO_3_	DMSO	33
12	Pd(PPh_3_)_4_	K_2_CO_3_	DMF	35
13	Pd(PPh_3_)_4_	K_2_CO_3_	CH_3_CN	0
14	Pd(PPh_3_)_4_	CuBr	Dioxane	33
15	Pd(PPh_3_)_4_	NH_4_Br	Dioxane	35
16	Pd(PPh_3_)_4_	TBAC	Dioxane	30
20	Pd(PPh_3_)_4_	TEA	Dioxane	trace
21	Pd(PPh_3_)_4_	Cs_2_CO_3_	Dioxane	42
22	Pd(PPh_3_)_4_	NaH	Dioxane	31
23	Pd(PPh_3_)_4_	NaOH	Dioxane	0
24	Pd(PPh_3_)_4_	t-BuOK	Dioxane	32
25^c^	Pd(PPh_3_)_4_	K_2_CO_3_	Dioxane	63
26^d^	Pd(PPh_3_)_4_	K_2_CO_3_	Dioxane	40

a Reaction conditions: **1a** (0.25 mmol), catalyst (5 mol%), base (0.5 mmol), and solvent at 80°C for 10 h under N_2_.

b Isolated yield.

c 100°C.

d 60°C.

Next, the reaction was carried out in different solvents such as toluene, EtOAc, dioxane, DMSO, DMF, and CH_3_CN to determine the optimal solvent (Table 1, entries 8–13). Subsequently, the effects of different additives such as CuBr, NH_4_Br, and TBAC, on the product yield were investigated. The product yield did not increase significantly in the presence of these additives (Table 1, entries 14 and 15). K_2_CO_3_ was the most effective in facilitating the reaction, while other bases such as TEA, Cs_2_CO_3_, NaH, NaOH, and t-BuOK led to significantly lower product yields. Increasing or decreasing the temperature had no significantly improve the reaction yield. (Table 1, entries 25, 26). Therefore, the optimal reaction conditions were **1a** (0.25 mmol) as the substrate, Pd(PPh_3_)_4_ as the catalyst (5 mol%), and K_2_CO_3_ (0.5 mmol) as the base in dioxane (0.1 M) for 10 h at 80°C under N_2_ conditions.

**SCHEME 2:**
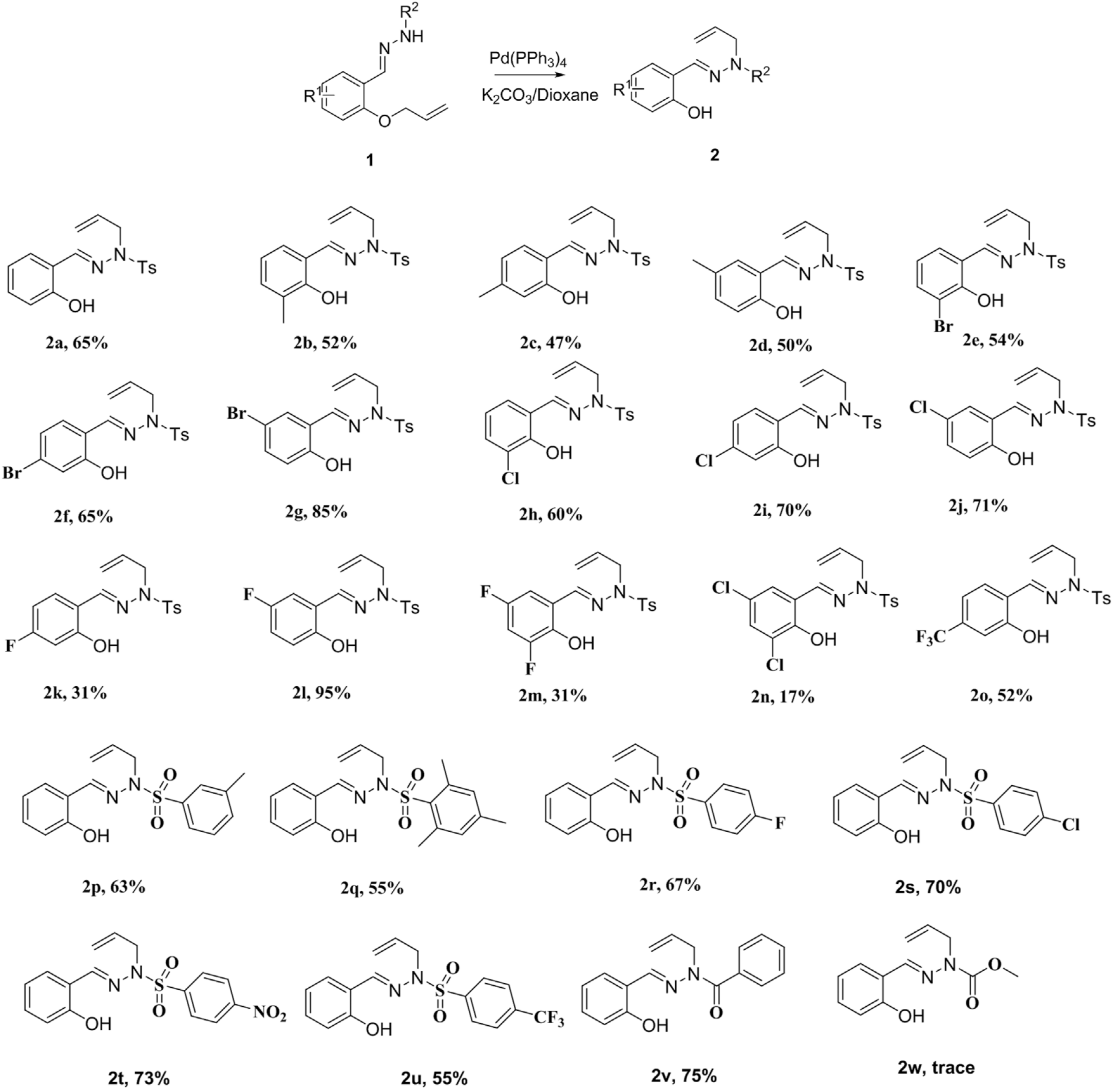
Scope of the reaction of *N*-sulfonylhydrazone. Reaction conditions: **1a** (0.25 mmol), catalyst (5 mol%), base (0.50 mmol), and solvent at 80°C for 10 h under N_2_.

With the optimal conditions in hand, we explored the scope of the reaction. First, we investigated the effect of various substituted *N*-sulfonylhydrazones as substrates (Scheme 2) on the reaction. The results revealed that the reaction conditions showed good tolerance for the functional groups on these substrates. Not only halogen groups (3-Br, 4-Br, 5-Br, 3-Cl, 4-Cl, 5-Cl, 4-F, 5-F, 3,5-2F, and 3,5-2Cl) and electron-donating substituents (3-CH_3_, 4-CH_3_, and 5-CH_3_) but also strongly electron-withdrawing (4-NO_2_, 4-CF_3_) groups could be tolerated under the optimized conditions, so that the reaction proceeded smoothly.

Halogen groups substituted at various positions on the benzene ring had different effects on the reaction. For example, halogen substitution at the 5-position of the benzene ring gave a higher yield (**2k–2l**) than did substitution at the 3**-** and 4-positions. In particular, 5-F substitution in the benzene ring generated the target compound in 95% isolated yield (**2l)**. However, the reaction yields were significantly lower when double halogen substitution was present on the phenyl ring (**2m**, **2n**). Moreover, the target compound (**2o**) was obtained smoothly when the substrate was charged with strong electron-withdrawing group (4-CF_3_), with yields of 52%.

Subsequently, we focused our attention on the effect of different substituted sulfonylhydrazones on the reaction yields (Scheme 2). The results showed that this method has wide applicability (**2p–2w**). Electron-withdrawing groups increased the yield of the reaction, while electron-donating groups decreased the yield. For example, the yields obtained with halogen substitution were higher than those observed with methyl substitution (**2p** and **2q** vs. **2r** and **2s**). Encouragingly, even with strong electron-withdrawing group substitution, the corresponding target compounds were furnished smoothly (**2t**, **2u**). The reaction also proceeded smoothly when the *p*-toluenesulfonyl group was replaced by the benzoyl group (**2v**), giving the target product in 75% yield. Unfortunately, the reaction did not proceed smoothly when the *p*-toluenesulfonate group was displaced by the methyl formate group (**2w**).

**FIGURE 1:**
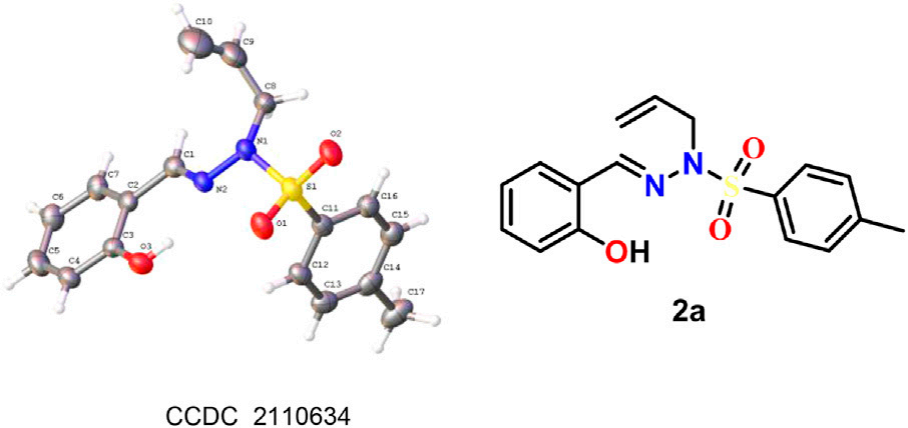
ORTEP diagram of compound **2a.**

**SCHEME 3:**
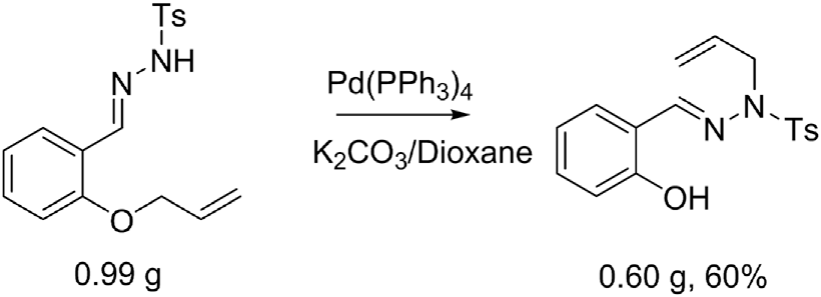
A scale-up experiment.

The reaction catalyzed by Pd(0) afforded sulfonylhydrazones, mainly the *trans*-isomer. The structure of **2a** was confirmed by X-ray single-crystal diffraction analysis, and the chemical structures of other examples were obtained by analogy (Figure 1, see Supplementary Material for details). Based on the above results, we performed a scale-up experiment to extend the adaptability of the reaction. When 7.0 mmol of **1a** was reacted under palladium catalysis, the corresponding product **2a** was obtained in 60% yield (Scheme 3).

**SCHEME 4:**
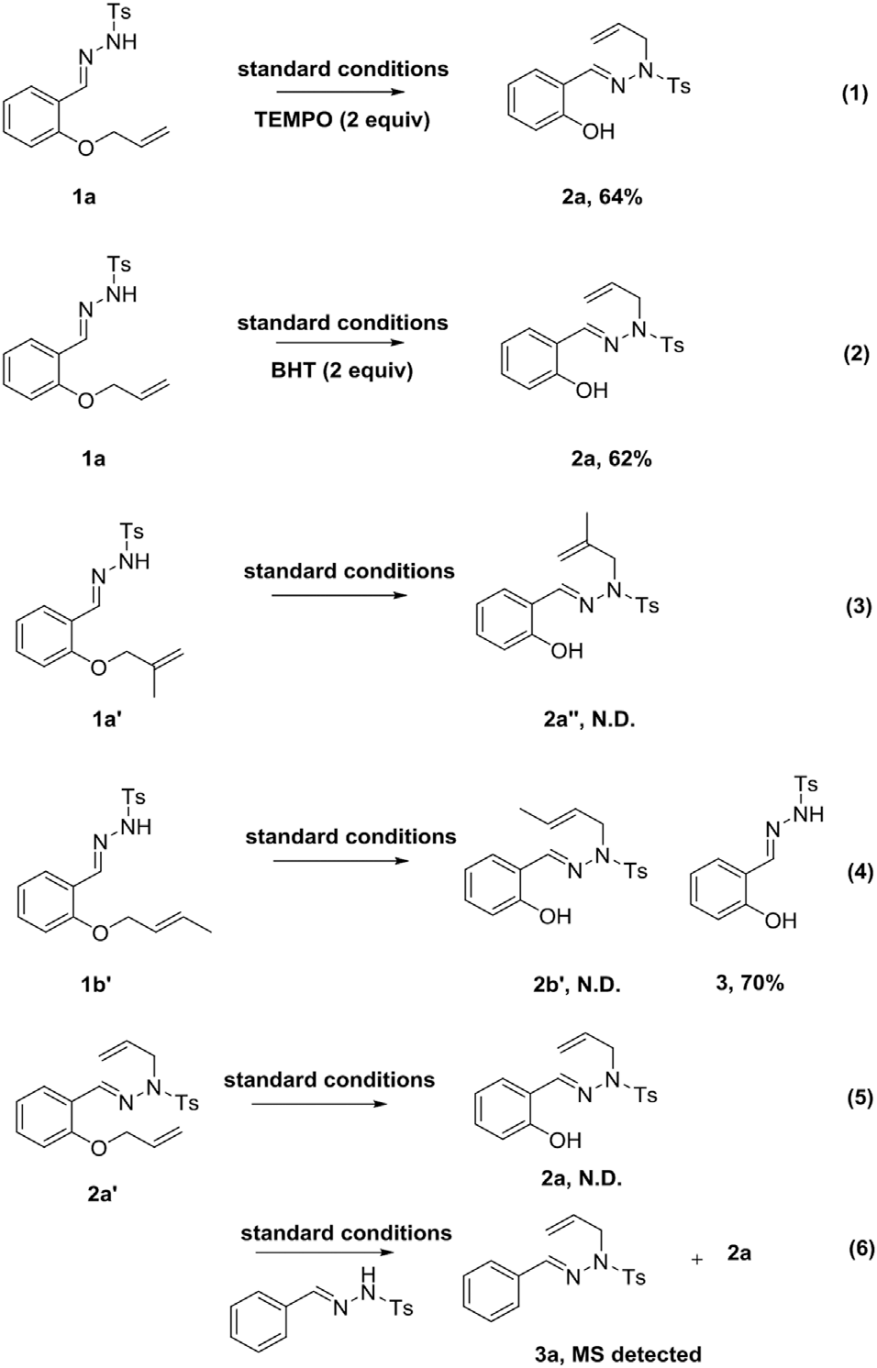
Controlled experiments.

Subsequently, the reaction mechanism was investigated. Upon introducing free radical inhibitors (TEMPO or BHT) into the system, the reaction proceeded smoothly to afford the corresponding products (Scheme 4, Eqs. 1 and 2). This result suggested that the reaction did not involve a free radical mechanism. Unfortunately, the reaction did not proceed smoothly when the allyl group was replaced by a 2-methylallyl group (Scheme 4, Eq. 3). When **1a** was substituted by substrate **1b′**, **2b′** was not obtained under standard conditions, but the compound **3** was afforded, indicating that the terminal double bond with substituent was easily removed in the reaction (Scheme 4, Eq. 4). When **1a** was replaced by substrate **2a′**, the target compound **2a** could not be obtained under the standard conditions (Scheme 4, Eq. 5). In contrast, if *N′*-benzylidene-4-methylbenzenesulfonyl hydrazide was added to the reaction system, **2a** and **3a** were produced (Scheme 4, Eq. 6).

**SCHEME 5:**
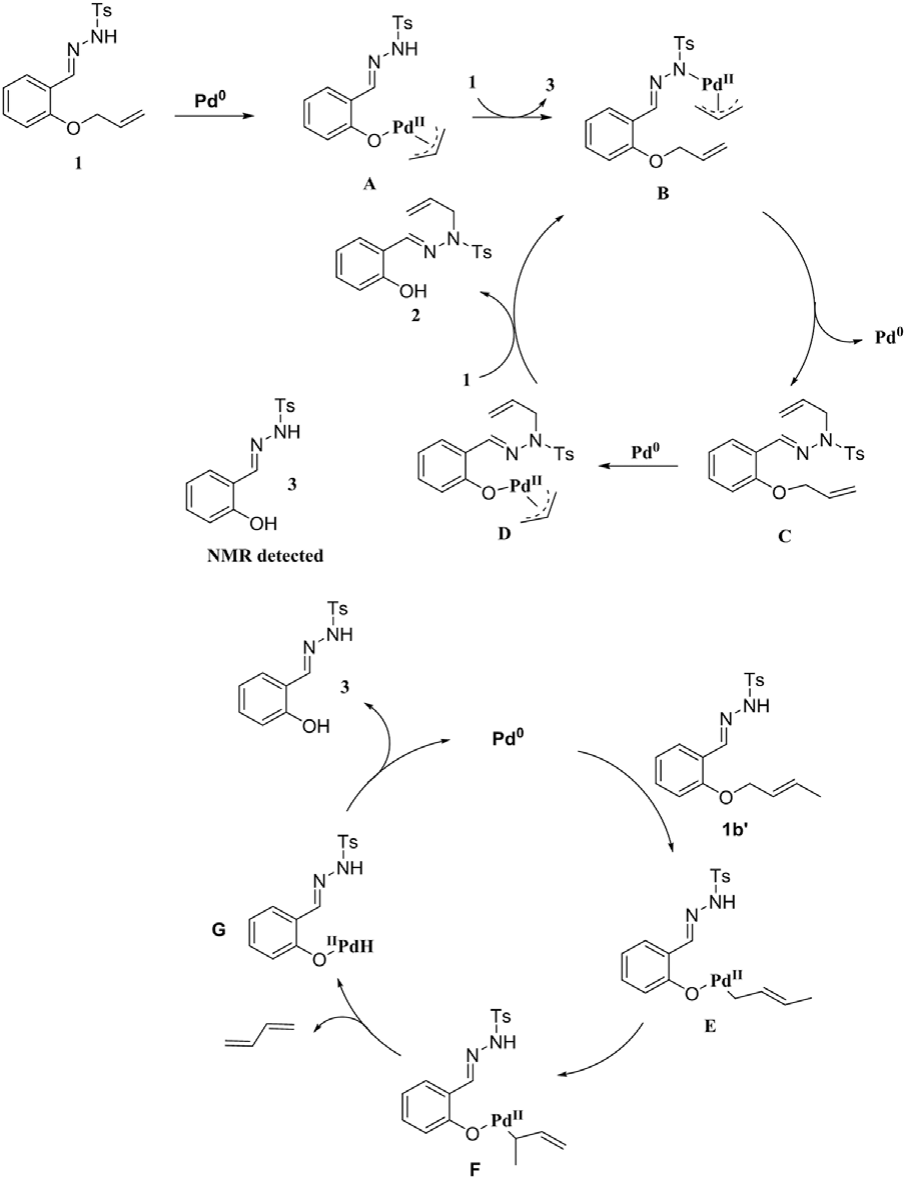
Proposed mechanism.

Based on these results and the literature reports, we propose a plausible reaction mechanism (Tang et al., 2021; He et al., 2019; Nakamura et al., 2007; Butt and Zhang, 2015; Huo et al., 2014; Nakamura et al., 2008; Liu et al., 2018; Ma and Jiao, 2002; Yamamoto and Radhak-rishnan, 1999; Sieber and Morken, 2006; Bates and Satcharoen, 2002; Hashmi et al., 2013; Kolundzič et al., 2014) (Scheme 5). Initially, **1** is added to Pd(0) *via* oxidation, followed by exchange with the ligand of **1** to give π-allylpalladium species **B**. Then, **B** undergoes reductive elimination to afford intermediate **C**, which reacts with Pd(0) to form intermediate **D**. Since there is no β-H atom, **D** is exchanged with molecule **1** to produce **B** and simultaneously generates the final product **2**. In addition, we also propose a possible reaction mechanism when the reaction substrate is **1b′**. Oxidative addition of **1b′** to Pd(0), isomerization and subsequent β-H elimination generate Pd-H species **F**. Reductive elimination of intermediate **F** to afford product **3** and release Pd(0) for the next cycle (Scheme 5).

## Conclusion

In conclusion, we report the palladium-catalyzed rearrangement of *N*-tosylhydrazones bearing allyl ethers to generate *trans*-olefin-substituted sulfonylhydrazones. We also investigated the applicability of the reaction to furnish the corresponding products, regardless of the presence of strongly electron-donating or electron-withdrawing substituents. The reaction involves the breakage of C-O bonds and the formation of C-N bonds, which forms the basis for the study of rearrangement reactions. Further investigation into the application of this reaction is ongoing in our laboratory.

## Data Availability

The datasets presented in this study can be found in online repositories. The names of the repository/repositories and accession number(s) can be found in the article/Supplementary Material.
