# Supplementary material for: Pd-Catalyzed Rearrangement Reaction of N-Tosylhydrazones Bearing Allyl Ethers Into Trans-Olefin-Substituted Sulfonylhydrazones
Source: Front Chem. 2021 Oct 25;9:782641. doi: 10.3389/fchem.2021.782641 (PMC8573317; doi:10.3389/fchem.2021.782641)
Supplement: Supplementary file 1 [file DataSheet1.pdf]

SUPPORTING INFORMATION

Pd-Catalyzed Rearrangement Reaction of *N*-Tosylhydrazones Bearing Allyl Ethers into *Trans*-Olefin-Substituted Sulfonylhydrazones

Yaoyao Chang,[a] Jianfang Fu,[a] Yingxue Li,[a] Rongcai Ding,[a] Yue Liu,*[a] Jinxing Hu*[a]

[a] Weifang Medical University , No.7166 Baotong Road, Weifang, 261053, PR China

Corresponding Author: * E-mail: liuyue@wfmc.edu.cn, jinxinghu2013@wfmc.edu.cn

Table of Contents

I. General Information……..S2

II. Spectral Data of Products..S3-S10

III. ^{1}H NMR and ^{13}C NMR Spectra..S11-S33

Ⅳ. X-Ray Crystallographic Data of 2a S34-S35

I. General Information

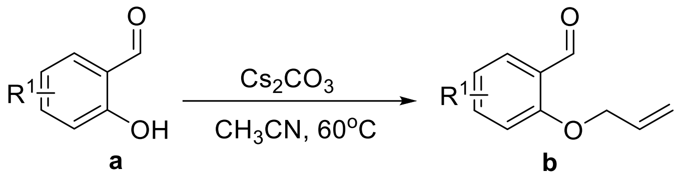

The round bottom flask was filled with aldehyde **a** (1 eq.), 3-bromopropene (1 eq.), Cs2CO3 (2 eq.) and CH_3CN. The reaction solution was stirred at elevated temperature to 60 °C and the progress of the reaction was monitored by TLC. After completion of the reaction, water was added and extracted with DCM for 3 times, the organic layers were combined, dried with anhydrous magnesium sulfate, and the solvent was evaporated to obtain the compound, which was used directly in the next reaction step without further purification.

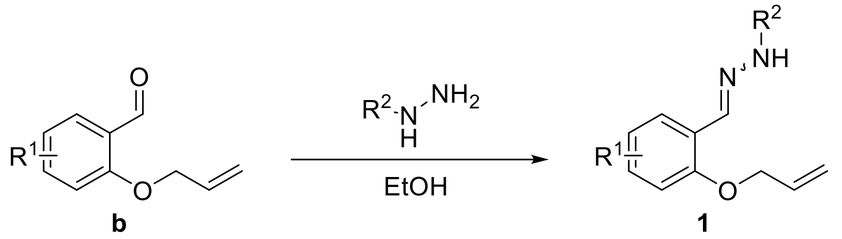

The reaction of **b** (1 eq.) with sulfonyl hydrazide (1.2 eq.) was added to ethanol solvent, 1 drop of acetic acid was added and the reaction was stirred at room temperature, after which completion was determined by TLC analysis. The solvent was removed, water was added and the solid was precipitated and purified by ether to give compound **1**.

(2) General procedure for the synthesis of products (2a as example).

Under a nitrogen atmosphere, **1a** (83 mg, 0.25 mmol) was added to dioxane (4 mL) solvent containing potassium carbonate (69 mg, 0.50 mmol, 2.0 equiv), [$Pd(PPh_3)_4$] (14.5 mg, 0.01 mmol, 5 mol%). The obtained mixture was stirred at 80 °C for 10 h. The reaction progress was monitored by TLC. After completion of the reaction, water (8 mL) was added, extracted with DCM, the organic layers were combined, and then the solvent was evaporated and the crude product was purified by thin layer chromatography (PE/EA=3:1) to obtain the target product **2a**.

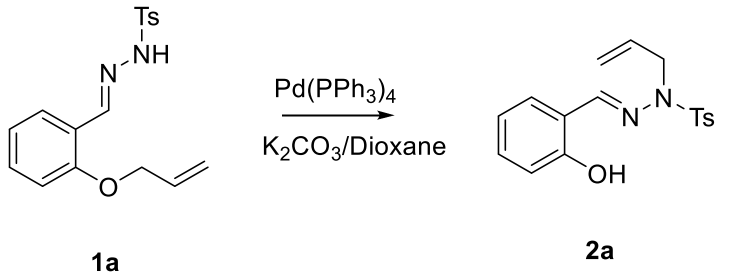

Ⅱ. Spectral Data of Products

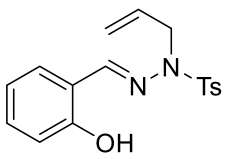

2a

***(E)-N-allyl-N'-(2-hydroxybenzylidene)-4-methylbenzenesulfonohydrazide* (2a)**

Yield: 65%, yellow liquid.^{1}H NMR (400 MHz,) δ 10.62 (s, 1H), 7.98 (s, 1H), 7.66 (m, 2H), 7.24 (m, 3H), 7.12 (d, *J* = 7.7 Hz, 1H), 6.89 (d, *J* = 8.3 Hz, 1H), 6.82 (t, *J* = 7.5 Hz, 1H), 5.70 (ddt, *J* = 15.9, 10.4, 5.3 Hz, 1H), 5.17 (m, 2H), 4.13 (d, *J* = 5.3 Hz, 2H), 2.35 (s, 3H). ^{13}C NMR (100 MHz, $CDCl_3$) δ 157.8, 153.9, 144.2, 132.7, 131.6, 131.0, 130.3, 129.3, 127.5, 118.8, 118.5, 116.8, 116.5, 50.4, 21.0 HRMS (ESI-Q-TOF, *m/z*) calcd for $C_{17}H_{18}N_2O_3S$ $[M+H]^+$: 331.1111, found $[M+H]^+$: 331.1102.

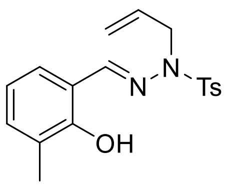

2b

***(E)-N-allyl-N'-(2-hydroxy-3-methylbenzylidene)-4-methylbenzenesulfonohydrazide* (2b)**

Yield: 52%, yellow liquid. ^{1}H NMR (400 MHz, Chloroform-*d*) δ 10.91 (s, 1H), 8.10 (s, 1H), 7.73 (d, *J* = 7.8 Hz, 2H), 7.33 (d, *J* = 7.9 Hz, 2H), 7.18 (d, *J* = 7.3 Hz, 1H), 7.06 (d, *J* = 7.7 Hz, 1H), 6.81 (t, *J* = 7.5 Hz, 1H), 5.76 (ddt, *J* = 15.9, 10.4, 5.1 Hz, 1H), 5.23 (m, 2H), 4.16 (d, *J* = 5.2 Hz, 2H), 2.43 (s, 3H), 2.28 (s, 3H). ^{13}C NMR (400 MHz, CDCl3) δ 10.9, 8.1, 7.7, 7.7, 7.3, 7.3, 7.2, 7.2, 7.1, 7.1, 6.8, 6.8, 6.8, 5.8, 5.8, 5.8, 5.8, 5.8, 5.8, 5.7, 5.7, 5.3, 5.2, 5.2, 4.2, 4.2, 2.4, 2.3. HRMS (ESI-Q-TOF, *m/z*) calcd for $C_{18}H_{20}N_2O_3S$ $[M+H]^+$: 345.1267, found $[M+H]^+$: 345.1260.

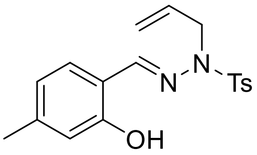

2c

***(E)-N-allyl-N'-(2-hydroxy-4-methylbenzylidene)-4-methylbenzenesulfonohydrazide* (2c)**

Yield: 47%, yellow liquid.^{1}H NMR (400 MHz, $CDCl_3$) δ 10.67 (s, 1H), 8.11 (s, 1H), 7.71 (d, *J* = 7.8 Hz, 2H), 7.32 (d, *J* = 7.9 Hz, 2H), 7.09 (d, *J* = 7.8 Hz, 1H), 6.73 (m, 2H), 5.76 (dq, *J* = 10.6, 5.5 Hz, 1H), 5.23 (d, *J* = 15.5 Hz, 2H), 4.13 (d, *J* = 5.3 Hz, 2H), 2.41 (s, 3H), 2.32 (s, 3H). ^{13}C NMR (100 MHz, $CDCl_3$) δ 158.7, 157.1, 144.7, 143.6, 142.9, 133.1, 131.7, 131.2, 129.9, 128.2, 120.7, 119.2, 117.5, 114.9, 51.8, 21.7, 21.6. HRMS (ESI-Q-TOF, *m/z*) calcd for $C_{18}H_{20}N_2O_3S$ $[M+H]^+$: 345.1267, found $[M+H]^+$: 345.1265.

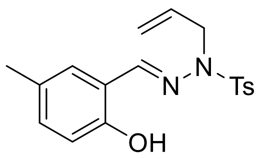

2d

***(E)-N-allyl-N'-(2-hydroxy-5-methylbenzylidene)-4-methylbenzenesulfonohydrazide* (2d)**
Yield: 50%, yellow liquid. ^{1}H NMR (400 MHz, $CDCl_3$) δ 10.48 (s, 1H), 8.04 (s, 1H), 7.72 (d, *J* = 7.8 Hz, 2H), 7.33 (d, *J* = 7.9 Hz, 2H), 7.11 (d, *J* = 8.3 Hz, 1H), 7.00 (s, 1H), 6.87 (d, *J* = 8.4 Hz, 1H), 5.76 (ddt, *J* = 15.9, 10.4, 5.3 Hz, 1H), 5.23 (dd, *J* = 13.3, 9.9 Hz, 2H), 4.18 (d, *J* = 5.2 Hz, 2H), 2.42 (s, 3H), 2.27 (s, 3H). ^{13}C NMR (100 MHz, $CDCl_3$) δ 156.4, 155.7, 144.7, 133.3, 133.2, 131.8, 131.0, 129.9, 128.6, 128.2, 119.2, 117.1, 116.9, 51.4, 21.6, 20.3. HRMS (ESI-Q-TOF, *m/z*) calcd for $C_{18}H_{20}N_2O_3S$ $[M+H]^+$: 345.1267, found $[M+H]^+$: 345.1267.

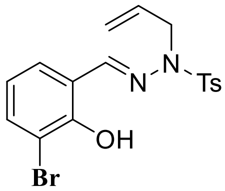

2e

***(E)-N-allyl-N'-(3-bromo-2-hydroxybenzylidene)-4-methylbenzenesulfonohydrazide* (2e)**
Yield: 54%, yellow solid, m.p.128.8-129.9°C. ^{1}H NMR (400 MHz, $CDCl_3$) δ 11.42 (s, 1H), 7.97 (d, *J* = 1.7 Hz, 1H), 7.74 (d, *J* = 7.8 Hz, 2H), 7.55 (d, *J* = 8.0 Hz, 1H), 7.34 (d, *J* = 7.9 Hz, 2H), 7.16 (d, *J* = 7.6 Hz, 1H), 6.79 (t, *J* = 7.9 Hz, 1H), 5.75 (ddt, *J* = 16.0, 10.5, 5.3 Hz, 1H), 5.25 (m, 2H), 4.24 (d, *J* = 5.2 Hz, 2H), 2.43 (s, 3H). ^{13}C NMR (100 MHz, $CDCl_3$) δ 154.9, 152.3, 145.0, 135.4, 133.4, 130.7, 130.6, 130.1, 128.0, 120.3, 119.3, 118.5, 111.0, 50.9, 21.7. HRMS (ESI-Q-TOF, *m/z*) calcd for $C_{17}H_{17}BrN_2O_3S$ $[M+H]^+$: 409.0216, 411.0196, found $[M+H]^+$: 409.0219, 411.0194.

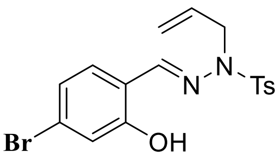

2f

***(E)-N-allyl-N'-(4-bromo-2-hydroxybenzylidene)-4-methylbenzenesulfonohydrazide* (2f)**
Yield: 65%, yellow liquid. ^{1}H NMR (400 MHz, $CDCl_3$) δ 10.87 (s, 1H), 7.93 (s, 1H), 7.72 (d, *J* = 7.8 Hz, 2H), 7.34 (d, *J* = 7.9 Hz, 2H), 7.15 (s, 1H), 7.02 (s, 2H), 5.76 (qd, *J* = 10.3, 5.1 Hz, 1H), 5.26 (d, *J* = 7.0 Hz, 1H), 5.23 (s, 1H), 4.23 (d, *J* = 4.9 Hz, 2H), 2.43 (s, 3H). ^{13}C NMR (100 MHz, $CDCl_3$) δ 158.9, 152.3, 144.9, 133.4, 132.3, 130.7, 130.0, 128.1, 126.0, 122.8, 120.5, 119.2, 116.5, 50.7, 21.7. HRMS (ESI-Q-TOF, *m/z*) calcd for $C_{17}H_{17}BrN_2O_3S$ $[M+H]^+$: 409.0216, 411.0196, found $[M+H]^+$: 409.0220, 411.0198.

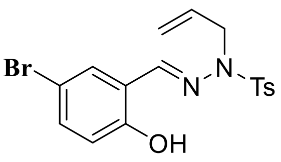

2g

***(E)-N-allyl-N'-(5-bromo-2-hydroxybenzylidene)-4-methylbenzenesulfonohydrazide* (2g)**
Yield: 85%, yellow solid, m.p.109.4-110.6°C. ^{1}H NMR (400 MHz, $CDCl_3$) δ 10.71 (s, 1H), 7.75 (m, 3H), 7.32 (d, *J* = 7.9 Hz, 3H), 6.84 (d, *J* = 8.8 Hz, 1H), 5.75 (ddd, *J* = 15.6, 10.2, 4.9 Hz, 1H), 5.26 (m, 2H), 4.31 (d, *J* = 4.2 Hz, 2H), 2.41 (s, 3H). ^{13}C NMR (100 MHz, $CDCl_3$) δ 157.1, 148.8, 145.0, 134.3, 133.7, 133.2, 130.4, 130.1, 127.9, 119.2, 119.1, 110.9, 50.0, 21.7. HRMS (ESI-Q-TOF, *m/z*) calcd for $C_{17}H_{17}BrN_2O_3S$ $[M+H]^+$: 409.0216, 411.0196, found $[M+H]^+$: 409.0218, 411.0196.

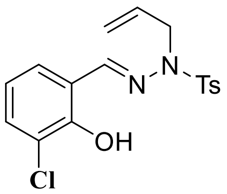

2h, 60%

***(E)-N-allyl-N'-(3-chloro-2-hydroxybenzylidene)-4-methylbenzenesulfonohydrazide*（2h）**
Yield: 60%, yellow solid. m.p.106.2-107.3°C. ^{1}H NMR (400 MHz, $CDCl_3$) δ 11.32 (s, 1H), 7.97 (s, 1H), 7.74 (d, *J* = 7.9 Hz, 2H), 7.36 (dd, *J* = 17.0, 7.9 Hz, 3H), 7.11 (d, *J* = 7.7 Hz, 1H), 6.85 (t, *J* = 7.8 Hz, 1H), 5.75 (qd, *J* = 10.3, 5.1 Hz, 1H), 5.25 (m, 2H), 4.25 (d, *J* = 4.8 Hz, 2H), 2.42 (s, 3H). ^{13}C NMR (100 MHz, $CDCl_3$) δ 154.0, 152.0, 145.0, 133.5, 132.3, 130.5, 130.1, 129.9, 128.0, 121.8, 119.8, 119.3, 118.7, 50.8, 21.6. HRMS (ESI-Q-TOF, *m/z*) calcd for $C_{17}H_{17}ClN_2O_3S$ $[M+H]^+$: 365.0721, found $[M+H]^+$: 365.0723.

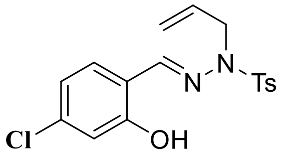

2i

***(E)-N-allyl-N'-(4-chloro-2-hydroxybenzylidene)-4-methylbenzenesulfonohydrazide* (2i)**
Yield: 70%, yellow solid, m.p.97.6-98.5°C. ^{1}H NMR (400 MHz, $CDCl_3$) δ 10.91 (s, 1H), 7.94 (s, 1H), 7.72 (d, *J* = 8.2 Hz, 2H), 7.33 (d, *J* = 8.0 Hz, 2H), 7.10 (d, *J* = 8.3 Hz, 1H), 6.97 (d, *J* = 1.6 Hz, 1H), 6.86 (dd, *J* = 8.2, 1.9 Hz, 1H), 5.76 (m, 1H), 5.25 (m, 2H), 4.24 (s, 2H), 2.42 (s, 3H). ^{13}C NMR (100 MHz, $CDCl_3$) δ 159.0, 152.1, 144.9, 137.7, 133.4, 132.2, 130.7, 130.0, 128.0, 119.9, 119.2, 117.5, 116.2, 50.7, 21.6. HRMS (ESI-Q-TOF, *m/z*) calcd for $C_{17}H_{17}ClN_2O_3S$ $[M+H]^+$: 365.0721, found $[M+H]^+$: 365.0723.

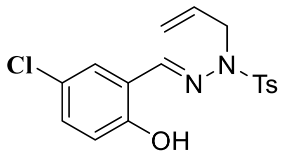

2j

(E)-N-allyl-N'-(5-chloro-2-hydroxybenzylidene)-4-methylbenzenesulfonohydrazide **（2j）**
Yield: 71%, yellow solid, m.p.104.0-105.2°C. ^{1}H NMR^{1}H NMR (400 MHz, $CDCl_3$) δ 10.69 (s, 1H), 7.78 (d, *J* = 7.0 Hz, 1H), 7.74 (d, *J* = 7.9 Hz, 2H), 7.31 (t, *J* = 7.3 Hz, 2H), 7.19 (d, *J* = 8.7 Hz, 1H), 7.13 (s, 1H), 6.89 (d, *J* = 8.8 Hz, 1H), 5.75 (ddd, *J* = 15.6, 10.2, 4.9 Hz, 1H), 5.26 (m, 2H), 4.31 (d, *J* = 4.5 Hz, 2H), 2.41 (s, 3H). ^{13}C NMR (100 MHz, $CDCl_3$) δ 156.6, 148.9, 145.0, 133.7, 131.5, 130.4, 130.3, 130.0, 127.9, 124.0, 119.1, 118.6, 118.6, 50.0, 21.6. HRMS (ESI-Q-TOF, *m/z*) calcd for $C_{17}H_{17}ClN_2O_3S$ $[M+H]^+$: 365.0721, found $[M+H]^+$: 365.0720.

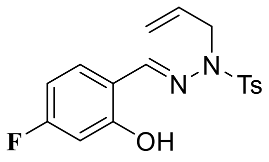

2k

(E)-N-allyl-N'-(4-fluoro-2-hydroxybenzylidene)-4-methylbenzenesulfonohydrazide **(2k)**
Yield: 31%, yellow liquid. ^{1}H NMR ^{1}H NMR (400 MHz, $CDCl_3$) δ 11.05 (s, 1H), 8.06 (d, *J* = 1.6 Hz, 1H), 7.71 (d, *J* = 7.9 Hz, 2H), 7.34 (d, *J* = 7.8 Hz, 2H), 7.17 (t, *J* = 7.5 Hz, 1H), 6.63 (m, 2H), 5.76 (ddt, *J* = 16.1, 10.6, 5.4 Hz, 1H), 5.24 (m, 2H), 4.17 (d, *J* = 5.3 Hz, 2H), 2.43 (s, 3H). ^{13}C NMR (100 MHz, $CDCl_3$) δ 166.4, 163.9, 160.7, 160.5, 154.9, 154.9, 144.9, 133.3, 133.2, 133.2, 130.9, 130.0, 128.2, 119.3, 114.2, 114.2, 107.4, 107.1, 104.6, 104.3, 51.4, 21.6. HRMS (ESI-Q-TOF, *m/z*) calcd for $C_{17}H_{17}FN_2O_3S$ $[M+H]^+$: 349.1017, found $[M+H]^+$: 349.1016.

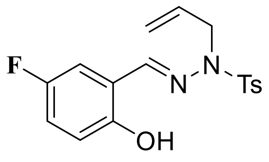

2l

(E)-N-allyl-N'-(5-fluoro-2-hydroxybenzylidene)-4-methylbenzenesulfonohydrazide **（2l）**
Yield: 95%, yellow liquid. ^{1}H NMR (400 MHz, $CDCl_3$) δ 10.51 (s, 1H), 7.80 (s, 1H), 7.74 (d, *J* = 7.9 Hz, 2H), 7.32 (d, *J* = 8.0 Hz, 2H), 6.96 (m, 1H), 6.88 (td, *J* = 8.4, 7.6, 3.5 Hz, 2H), 5.76 (ddt, *J* = 15.8, 10.2, 5.0 Hz, 1H), 5.26 (m, 2H), 4.31 (d, *J* = 4.6 Hz, 2H), 2.40 (s, 3H). ^{13}C NMR (100 MHz, $CDCl_3$) δ 156.9, 154.6, 154.2, 154.2, 149.2, 149.2, 145.0, 133.7, 130.4, 130.1, 127.9, 119.0, 118.7, 118.5, 118.2, 118.1, 117.6, 117.6, 116.6, 116.3, 50.0, 21.6. HRMS (ESI-Q-TOF, *m/z*) calcd for $C_{17}H_{17}FN_2O_3S$ $[M+H]^+$: 349.1017, found $[M+H]^+$: 349.1020.

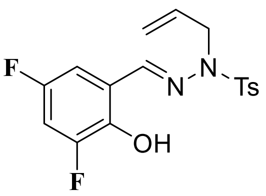

2m

(E)-N-allyl-N'-(3,5-difluoro-2-hydroxybenzylidene)-4-methylbenzenesulfonohydrazide **（2m）**

Yield: 31%, yellow liquid. ^{1}H NMR (400 MHz, $CDCl_3$) δ 7.76 (t, *J* = 7.5 Hz, 3H), 7.34 (d, *J* = 7.9 Hz, 3H), 5.78 (tt, *J* = 14.1, 6.7 Hz, 1H), 5.30 (m, 2H), 3.79 (d, *J* = 7.4 Hz, 2H), 2.45 (s, 3H). ^{13}C NMR (100 MHz, $CDCl_3$) δ 147.3, 145.1, 144.8, 135.4, 133.7, 130.1, 130.1, 129.9, 129.7, 128.5, 127.9, 127.6, 124.8, 124.6, 119.2, 111.5, 111.5 111.3 111.2, 106.8, 106.6, 106.6, 106.4, 61.0, 49.7, 21.6. HRMS (ESI-Q-TOF, *m/z*) calcd for $C_{17}H_{16}F_2N_2O_3S$ $[M+H]^+$: 367.0922, found $[M+H]^+$: 367.0922.

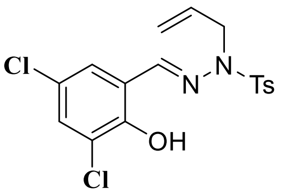

2n

(E)-N-allyl-N'-(3,5-dichloro-2-hydroxybenzylidene)-4-methylbenzenesulfonohydrazide **（2n）**

Yield: 17%, yellow liquid. ^{1}H NMR (400 MHz, $CDCl_3$) δ 7.67 (d, *J* = 7.3 Hz, 3H), 7.27 (d, *J* = 7.8 Hz, 3H), 5.71 (dq, *J* = 16.9, 8.2 Hz, 1H), 5.25 (d, *J* = 10.1 Hz, 1H), 5.08 (d, *J* = 17.1 Hz, 1H), 3.72 (d, *J* = 7.3 Hz, 2H), 2.37 (s, 3H). ^{13}C NMR (100 MHz, $CDCl_3$) δ 151.4, 146.1, 143.7, 134.4, 130.3, 129.1, 129.0, 128.8, 128.7, 127.8, 127.5, 126.8, 126.6, 123.8, 123.5, 118.3, 118.2, 59.9, 20.6. HRMS (ESI-Q-TOF, *m/z*) calcd for $C_{17}H_{16}Cl_2N_2O_3S$ $[M+H]^+$: 399.0331 found $[M+H]^+$: 399.0329.

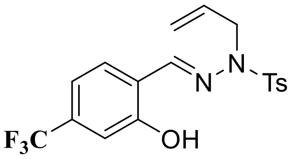

2o

(E)-N-allyl-N'-(2-hydroxy-4-(trifluoromethyl)benzylidene)-4-methylbenzenesulfonohydrazide **（2o）**

Yield: 52%, yellow solid, m.p.108.0-109.1°C. ^{1}H NMR (400 MHz, $CDCl_3$) δ 10.90 (s, 1H), 7.88 (s, 1H), 7.76 (d, *J* = 7.8 Hz, 2H), 7.34 (d, *J* = 7.9 Hz, 2H), 7.26 (m, 1H), 7.22 (s, 1H), 7.11 (d, *J* = 8.0 Hz, 1H), 5.77 (ddd, *J* = 15.6, 10.1, 5.0 Hz, 1H), 5.28 (m, 2H), 4.34 (d, *J* = 4.5 Hz, 2H), 2.43 (s, 3H). ^{13}C NMR (100 MHz, $CDCl_3$) δ 158.1, 148.5, 145.1, 133.7, 131.5, 130.3, 130.1, 127.9, 120.2, 119.1, 115.9, 115.9, 115.9, 115.8, 114.6, 114.5, 114.5, 114.5, 49.9, 21.6. HRMS (ESI-Q-TOF, *m/z*) calcd for $C_{18}H_{17}F_3N_2O_3S$ $[M+H]^+$: 399.0985, found $[M+H]^+$: 399.0984.

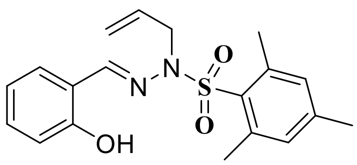

2q

***(E)-N-allyl-N'-(2-hydroxybenzylidene)-2,4,6-trimethylbenzenesulfonohydrazide*（2q）**

Yield: 55%, yellow solid, m.p.120.6-121.4°C. ^{1}H NMR (400 MHz, $CDCl_3$) δ 9.94 (s, 1H), 7.71 (s, 1H), 7.21 (q, *J* = 9.6, 7.8 Hz, 1H), 7.10 (d, *J* = 7.6 Hz, 1H), 7.00 (s, 2H), 6.84 (m, 2H), 5.88 (ddt, *J* = 15.7, 10.0, 4.8 Hz, 1H), 5.35 (m, 2H), 4.60 (d, *J* = 4.4 Hz, 2H), 2.64 (s, 6H), 2.30 (s, 3H).^{13}C NMR (100 MHz, $CDCl_3$) δ 157.5, 146.1, 143.8, 140.9, 132.3, 131.3, 131.0, 130.8, 130.6, 119.4, 119.2, 117.7, 117.0, 46.7, 22.9, 21.1. HRMS (ESI-Q-TOF, *m/z*) calcd for $C_{19}H_{22}N_2O_3S$ $[M+H]^+$: 359.1424 , found $[M+H]^+$: 359.1423.

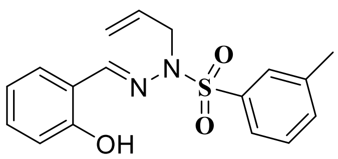

2p

***(E)-N-allyl-N'-(2-hydroxybenzylidene)-2-methylbenzenesulfonohydrazide*（2p）**

Yield: 63%, yellow liquid. ^{1}H NMR (400 MHz, $CDCl_3$) δ 10.70 (s, 1H), 8.04 (s, 1H), 7.73 (d, *J* = 7.9 Hz, 2H), 7.31 (dd, *J* = 16.7, 8.0 Hz, 3H), 7.19 (d, *J* = 7.6 Hz, 1H), 6.96 (d, *J* = 8.3 Hz, 1H), 6.89 (t, *J* = 7.5 Hz, 1H), 5.77 (ddt, *J* = 15.9, 10.4, 5.2 Hz, 1H), 5.24 (m, 2H), 4.21 (d, *J* = 5.1 Hz, 2H), 2.41 (s, 3H). ^{13}C NMR (100 MHz, $CDCl_3$) δ 158.5, 154.5, 144.8, 133.4, 132.2, 131.7, 130.9, 130.0, 128.1, 119.5, 119.1, 117.4, 117.2, 51.1, 21.6. HRMS (ESI-Q-TOF, *m/z*) calcd for $C_{17}H_{18}N_2O_3S$ $[M+H]^+$: 331.1111, found $[M+H]^+$: 331.1110.

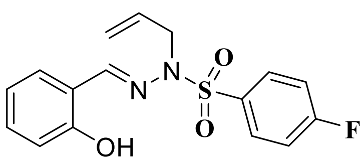

2r

***(E)-N-allyl-4-fluoro-N'-(2-hydroxybenzylidene)benzenesulfonohydrazide*（2r）**

Yield: 67%, yellow liquid. ^{1}H NMR (400 MHz, $CDCl_3$) δ 10.32 (s, 1H), 8.00 (t, *J* = 7.4 Hz, 1H), 7.91 (s, 1H), 7.61 (dt, *J* = 14.3, 7.0 Hz, 1H), 7.33 (dd, *J* = 13.9, 6.6 Hz, 1H), 7.25 (m, 2H), 7.17 (m, 1H), 6.89 (m, 2H), 5.87 (ddd, *J* = 15.7, 10.0, 4.8 Hz, 1H), 5.32 (dd, *J* = 21.6, 13.7 Hz, 2H), 4.52 (d, *J* = 4.2 Hz, 2H). ^{13}C NMR (100 MHz, $CDCl_3$) δ 160.2, 158.1, 157.7, 150.5, 136.2, 136.1, 130.0, 131.9, 131.4, 130.8, 125.2, 125.0, 124.8, 124.8, 119.4, 118.8, 117.6, 117.4, 117.2, 49.7, 49.7. HRMS (ESI-Q-TOF, *m/z*) calcd for $C_{16}H_{15}FN_2O_3S$ $[M+H]^+$: 335.0860, found $[M+H]^+$: 335.0860.

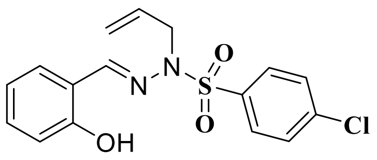

2s

***(E)-N-allyl-4-chloro-N'-(2-hydroxybenzylidene)benzenesulfonohydrazide*（2s）**

Yield: 70%, yellow liquid. ^{1}H NMR (400 MHz, $CDCl_3$) δ 10.60 (s, 1H), 8.04 (s, 1H), 7.79 (d, *J* = 8.2 Hz, 2H), 7.51 (d, *J* = 8.2 Hz, 2H), 7.31 (t, *J* = 7.8 Hz, 1H), 7.20 (d, *J* = 7.6 Hz, 1H), 6.97 (d, *J* = 8.3 Hz, 1H), 6.90 (t, *J* = 7.5 Hz, 1H), 5.76 (ddt, *J* = 15.9, 10.3, 5.2 Hz, 1H), 5.26 (m, 2H), 4.23 (d, *J* = 5.0 Hz, 2H). ^{13}C NMR (100 MHz, $CDCl_3$) δ 158.5, 155.0, 140.5, 134.9, 132.5, 131.8, 130.5, 129.7, 129.5, 119.6, 119.4, 117.2, 50.9. HRMS (ESI-Q-TOF, *m/z*) calcd for $C_{16}H_{15}ClN_2O_3S$ $[M+H]^+$: 351.0565, found $[M+H]^+$: 351.0563.

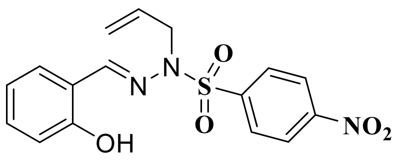

2t

***(E)-N-allyl-N'-(2-hydroxybenzylidene)-4-nitrobenzenesulfonohydrazide*（2t）**

Yield: 73%, yellow solid, m.p.132.3-133.4°C. ^{1}H NMR (400 MHz, $CDCl_3$) δ 10.44 (s, 1H), 8.40 (d, *J* = 8.2 Hz, 2H), 8.07 (m, 3H), 7.35 (t, *J* = 7.8 Hz, 1H), 7.22 (d, *J* = 7.7 Hz, 1H), 6.99 (d, *J* = 8.3 Hz, 1H), 6.93 (t, *J* = 7.5 Hz, 1H), 5.77 (ddt, *J* = 16.0, 10.5, 5.4 Hz, 1H), 5.27 (m, 2H), 4.28 (d, *J* = 5.1 Hz, 2H). ^{13}C NMR (100 MHz, $CDCl_3$) δ 158.5, 156.1, 150.7, 142.0, 132.9, 131.9, 130.1, 129.4, 124.5, 119.9, 119.8, 117.3, 117.0, 51.0. HRMS (ESI-Q-TOF, *m/z*) calcd for $C_{16}H_{15}N_3O_5S$ $[M+H]^+$: 362.0805, found $[M+H]^+$: 362.0802.

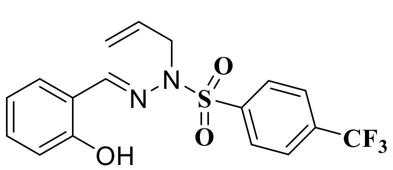

2u

***(E)-N-allyl-N'-(2-hydroxybenzylidene)-4-(trifluoromethyl)benzenesulfonohydrazide*(2u)**

Yield: 55%, yellow liquid. ^{1}H NMR (400 MHz, $CDCl_3$) δ 10.54 (s, 1H), 8.07 (s, 1H), 7.99 (d, *J* = 8.0 Hz, 2H), 7.82 (d, *J* = 8.0 Hz, 2H), 7.33 (t, *J* = 7.8 Hz, 1H), 7.21 (d, *J* = 7.7 Hz, 1H), 6.98 (d, *J* = 8.3 Hz, 1H), 6.91 (t, *J* = 7.5 Hz, 1H), 5.77 (ddd, *J* = 21.8, 10.4, 5.2 Hz, 1H), 5.27 (m, 2H), 4.26 (d, *J* = 5.0 Hz, 2H). ^{13}C NMR (100 MHz, $CDCl_3$) δ 158.5, 155.4, 140.0, 135.5, 135.2, 132.7, 131.8, 130.3, 129.2, 128.6, 126.5, 126.5, 126.5, 126.4, 124.5, 121.7, 119.7, 119.6, 117.3, 117.1, 50.9. HRMS (ESI-Q-TOF, *m/z*) calcd for $C_{17}H_{15}F_3N_2O_3S$ $[M+H]^+$: 385.0828, found $[M+H]^+$: 385.0830.

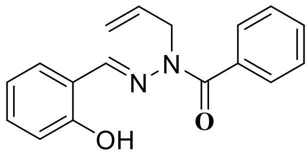

2v

***(E)-N-allyl-N'-(2-hydroxybenzylidene)benzohydrazide*(2v)**

Yield: 75%, yellow liquid. ^{1}H NMR (400 MHz, $CDCl_3$) δ 9.64 (s, 1H), 7.82 (s, 1H), 7.57 (d, *J* = 7.5 Hz, 2H), 7.50 (dq, *J* = 13.9, 6.9 Hz, 3H), 7.19 (m, 2H), 6.84 (dd, *J* = 18.6, 7.9 Hz, 2H), 5.90 (ddd, *J* = 15.3, 9.6, 4.4 Hz, 1H), 5.28 (m, 2H), 4.82 (d, *J* = 2.2 Hz, 2H). ^{13}C NMR (100 MHz, $CDCl_3$) δ 170.9, 157.2, 144.1, 134.9, 131.5, 131.3, 130.7, 130.0, 128.6, 127.8, 119.5, 117.7, 117.6, 117.0, 43.4. HRMS (ESI-Q-TOF, *m/z*) calcd for $C_{17}H_{16}N_2O_2$ [M+H]+: 281.1285, found [M+H]+: 281.1286.

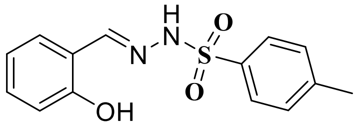

3

***(E)-N'-(2-hydroxybenzylidene)-4-methylbenzenesulfonohydrazide*(3)**

Yield: 15%, yellow liquid. ^{1}H NMR (400 MHz, $CDCl_3$) δ 8.42 (s, 1H), 7.88 (d, *J* = 8.3 Hz, 2H), 7.78 (s, 1H), 7.56 (m, 2H), 7.31 (m, 5H), 2.39 (s, 3H). ^{13}C NMR (100 MHz, $CDCl_3$) δ 148.0, 144.3, 135.3, 133.2, 130.4, 129.7, 128.6, 128.0, 127.4, 21.6. HRMS (ESI-Q-TOF, *m/z*) calcd for $C_{14}H_{14}N_2O_3S$ $[M+H]^+$: 291.0798, found $[M+H]^+$: 291.0802.

Ⅲ. ^{1}H NMR and ^{13}C NMR Spectra

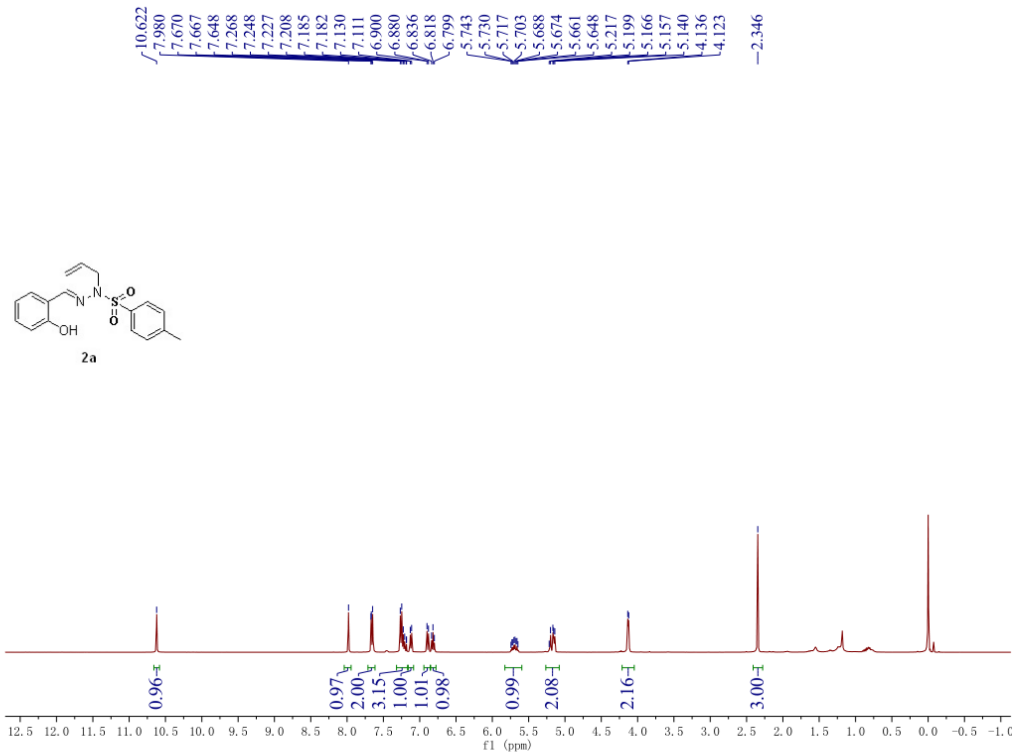

^{1}H NMR spectrum of compound **2a** (400 MHz, $CDCl_3$)

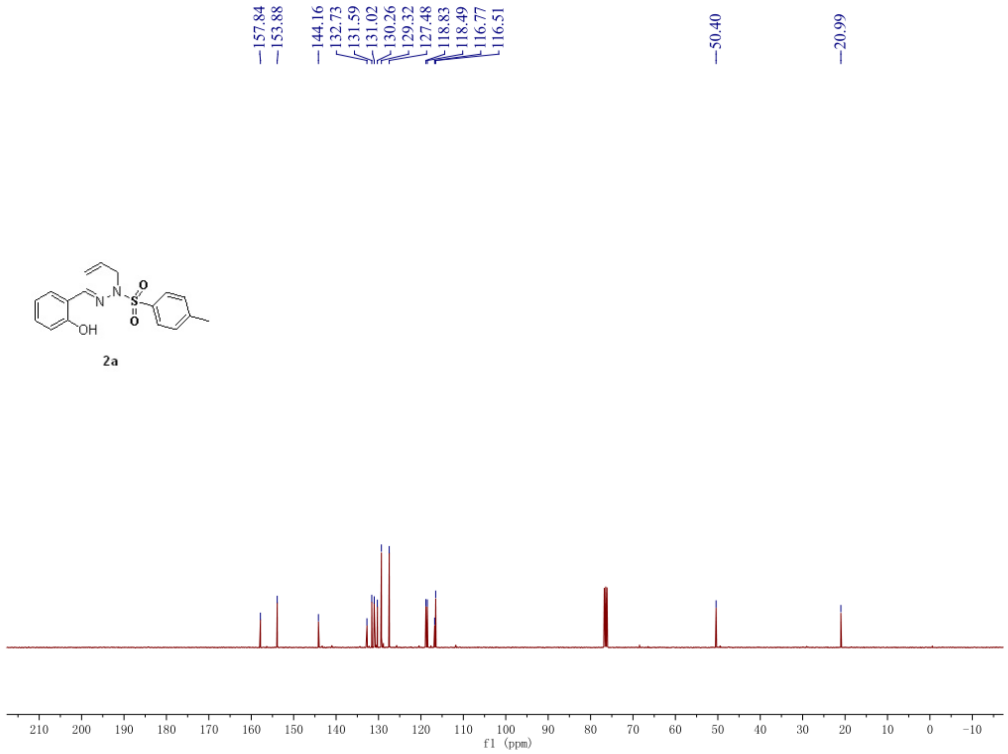

^{13}C NMR spectrum of compound **2a** (100 MHz, $CDCl_3$)

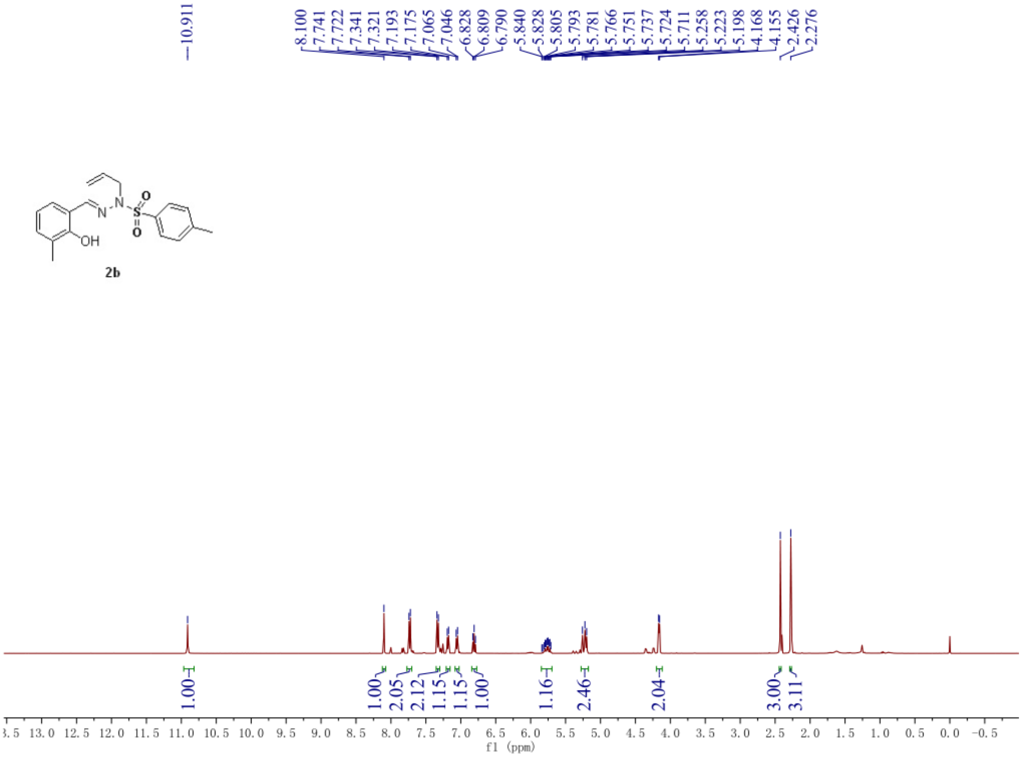

^{1}H NMR spectrum of compound **2b** (400 MHz, $CDCl_3$)

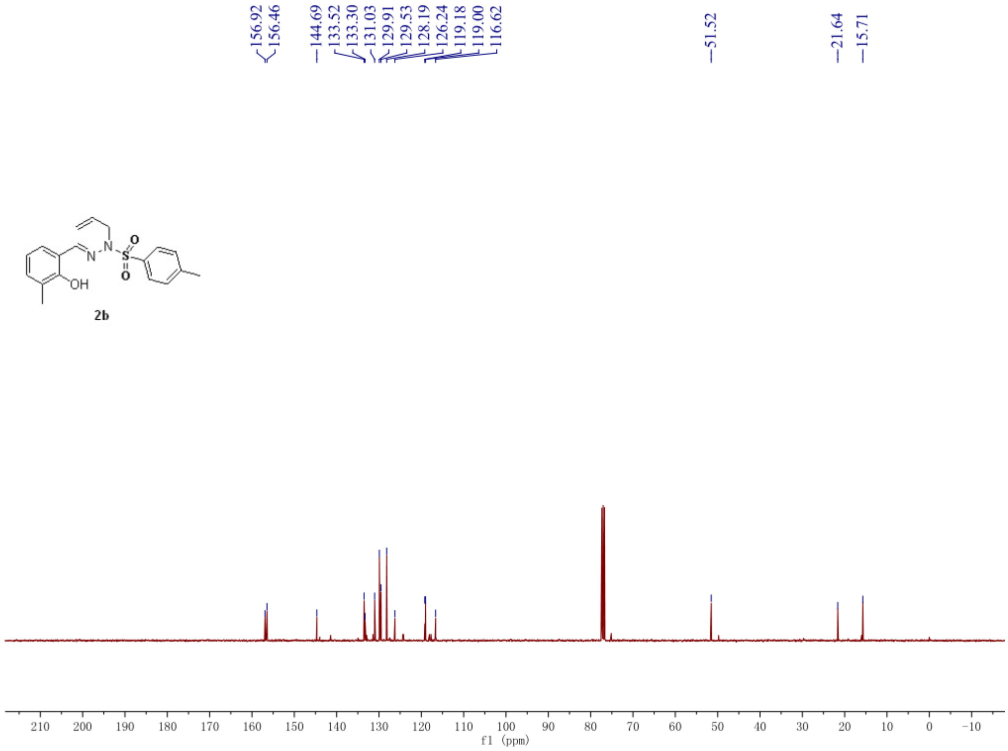

^{13}C NMR spectrum of compound **2b** (100 MHz, $CDCl_3$)

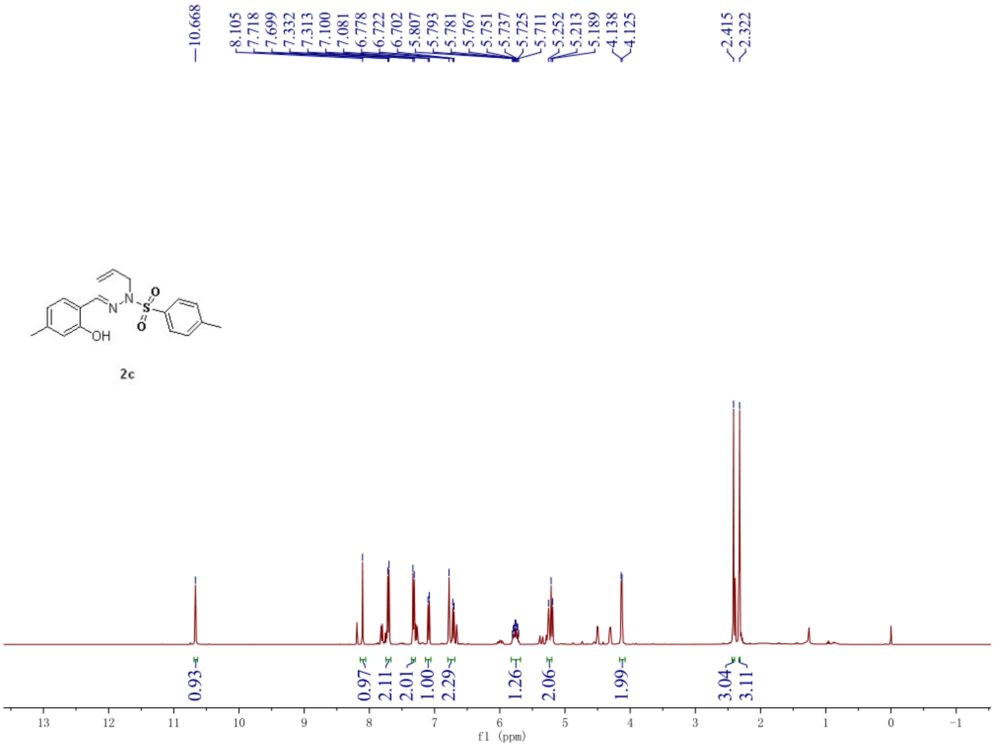

^{1}H NMR spectrum of compound **2c** (400 MHz, $CDCl_3$)

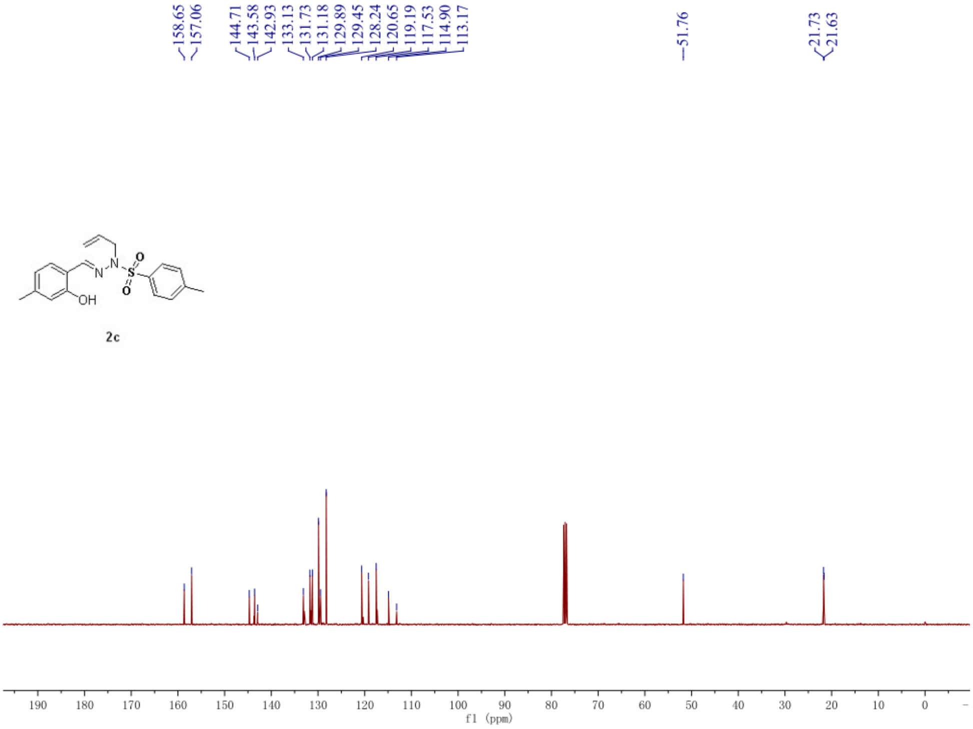

^{13}C NMR spectrum of compound **2c** (100 MHz, $CDCl_3$)

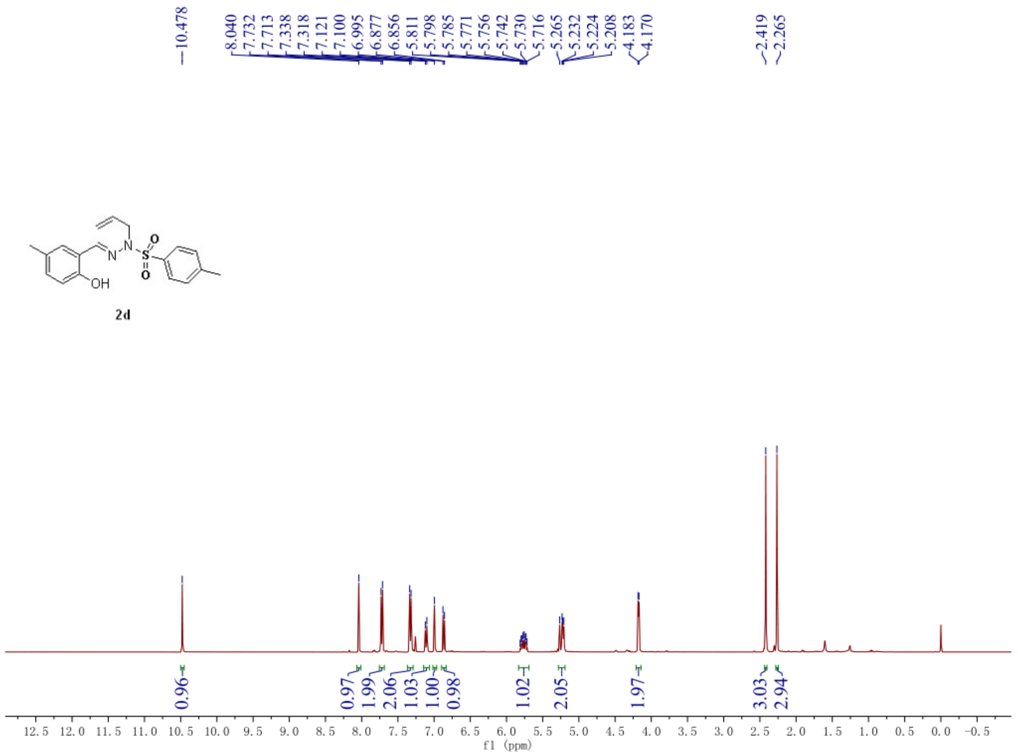

^{1}H NMR spectrum of compound **2d** (400 MHz, $CDCl_3$)

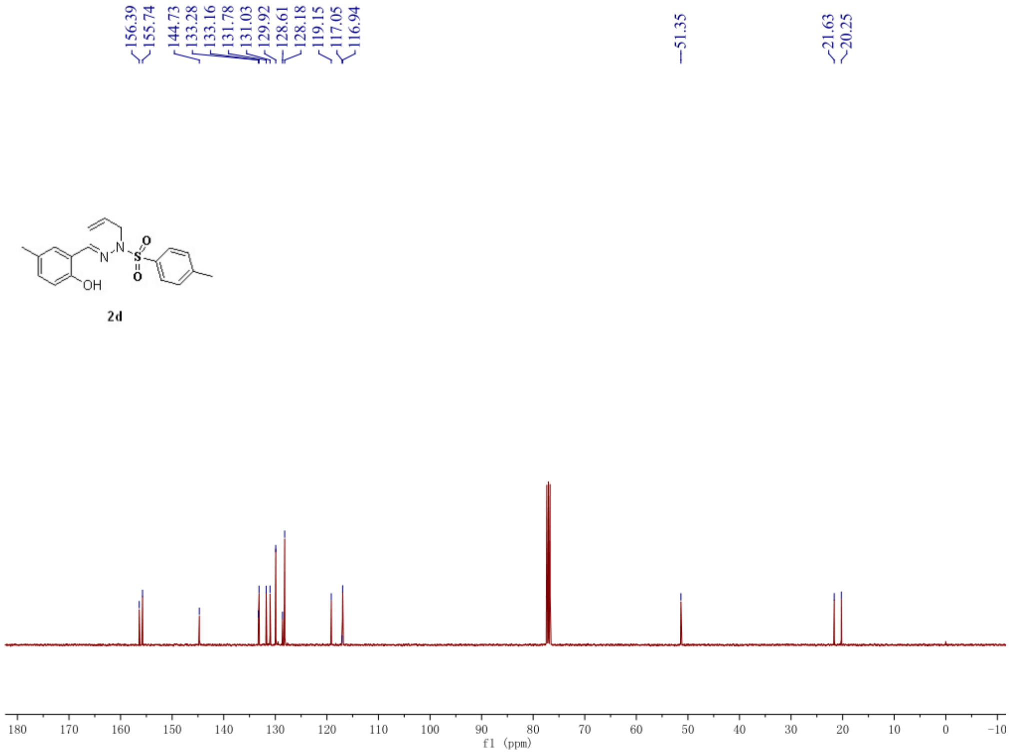

^{13}C NMR spectrum of compound **2d** (100 MHz, $CDCl_3$)

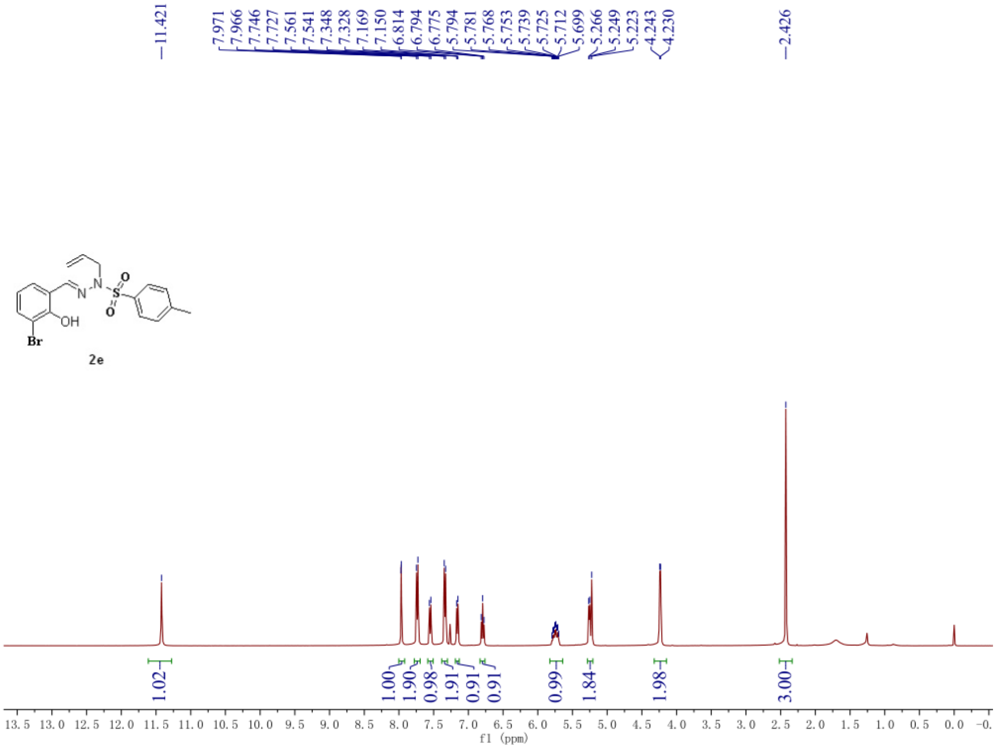

^{1}H NMR spectrum of compound **2e** (400 MHz, $CDCl_3$)

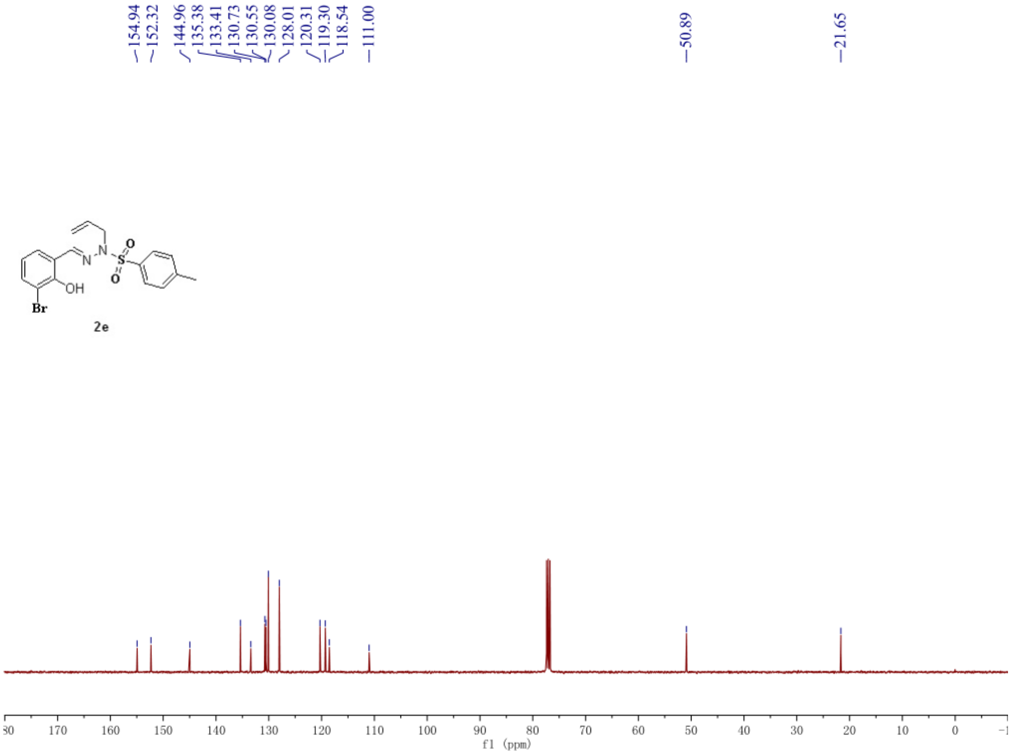

^{13}C NMR spectrum of compound **2e** (100 MHz, $CDCl_3$)

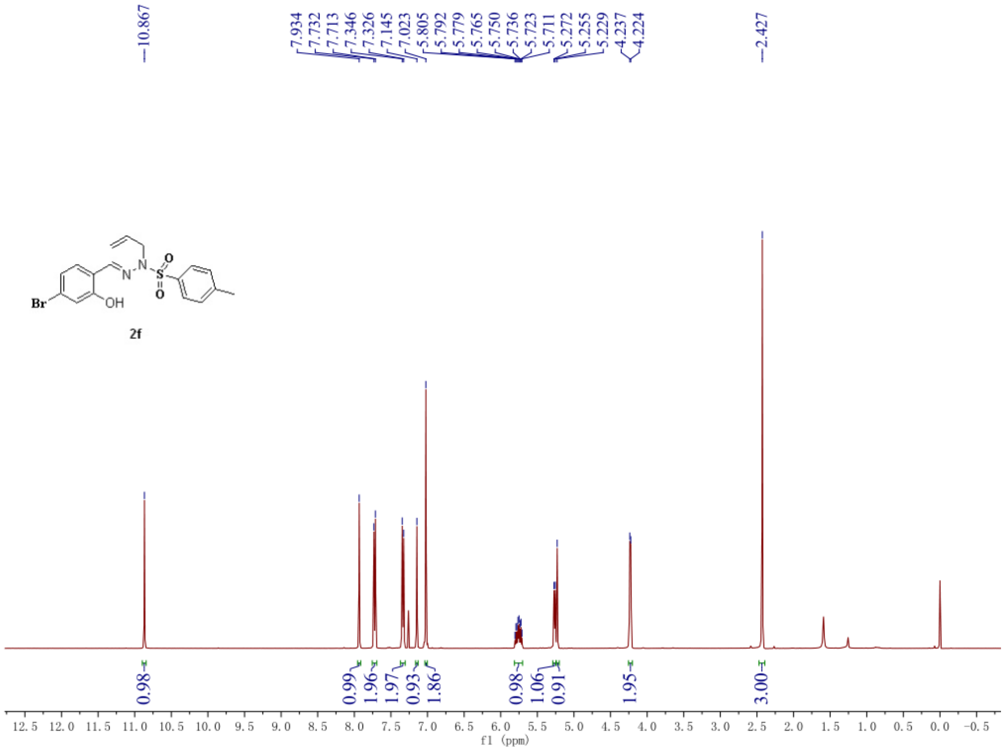

^{1}H NMR spectrum of compound **2f** (400 MHz, $CDCl_3$)

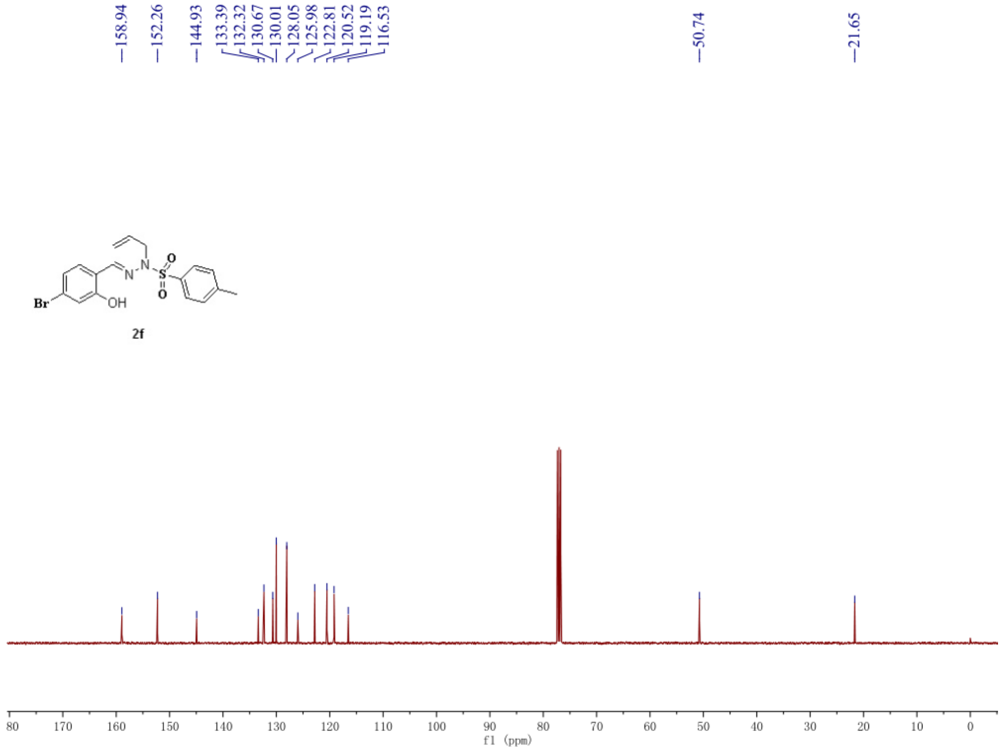

^{13}C NMR spectrum of compound **2f** (100 MHz, $CDCl_3$)

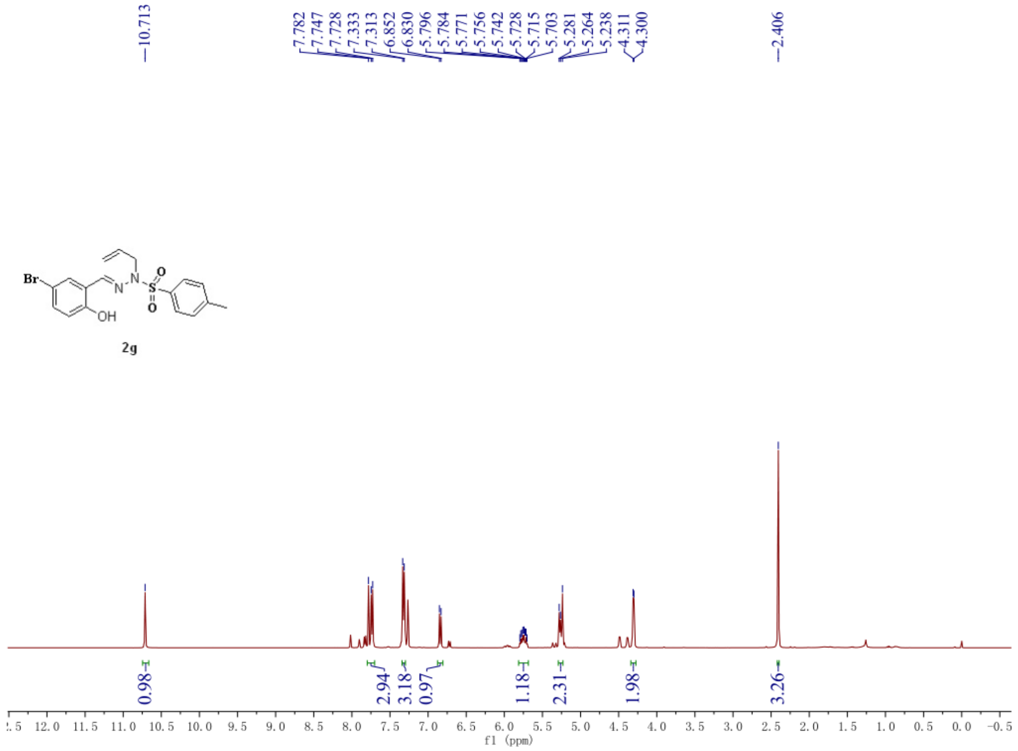

^{1}H NMR spectrum of compound **2g** (400 MHz, $CDCl_3$)

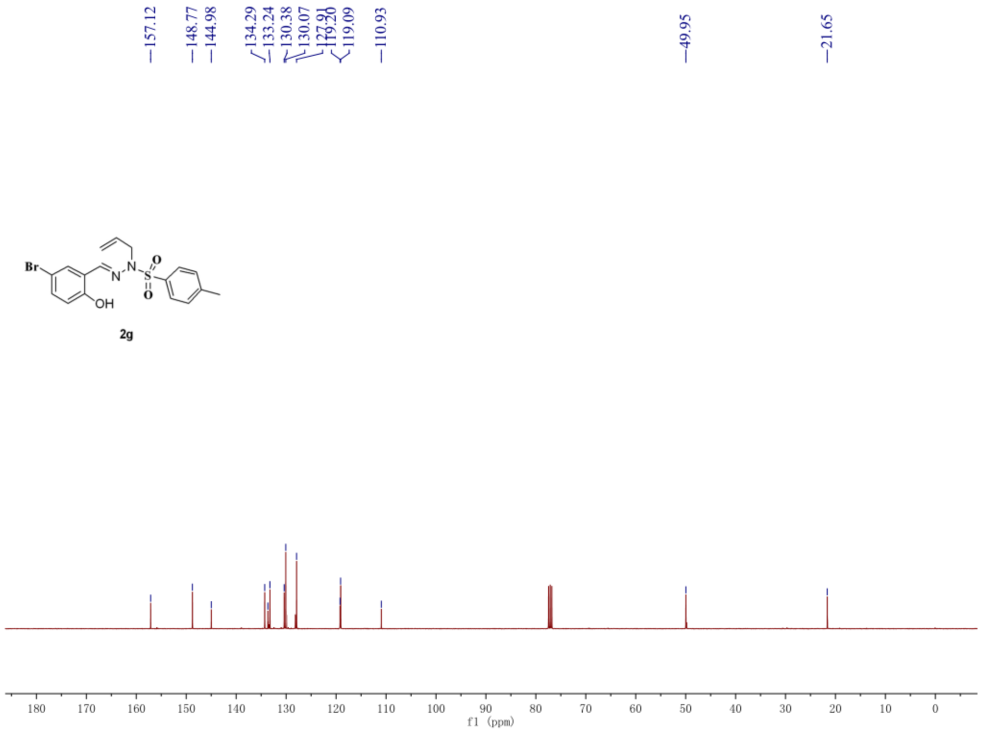

^{13}C NMR spectrum of compound **2g** (100 MHz, $CDCl_3$)

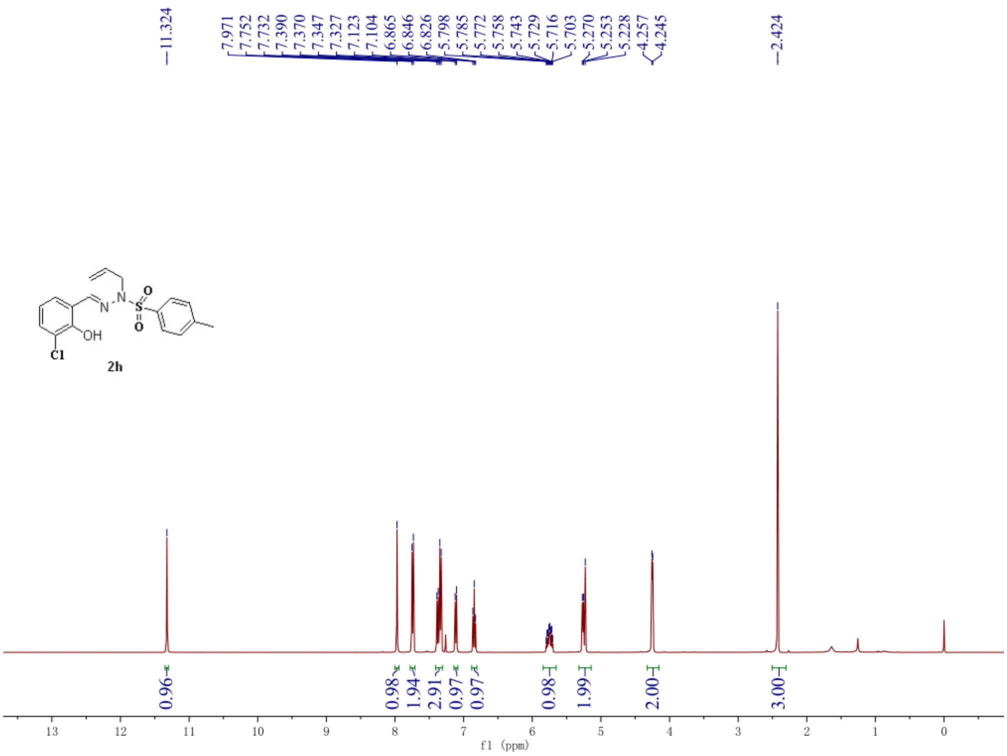

^{1}H NMR spectrum of compound **2h** (400 MHz, $CDCl_3$)

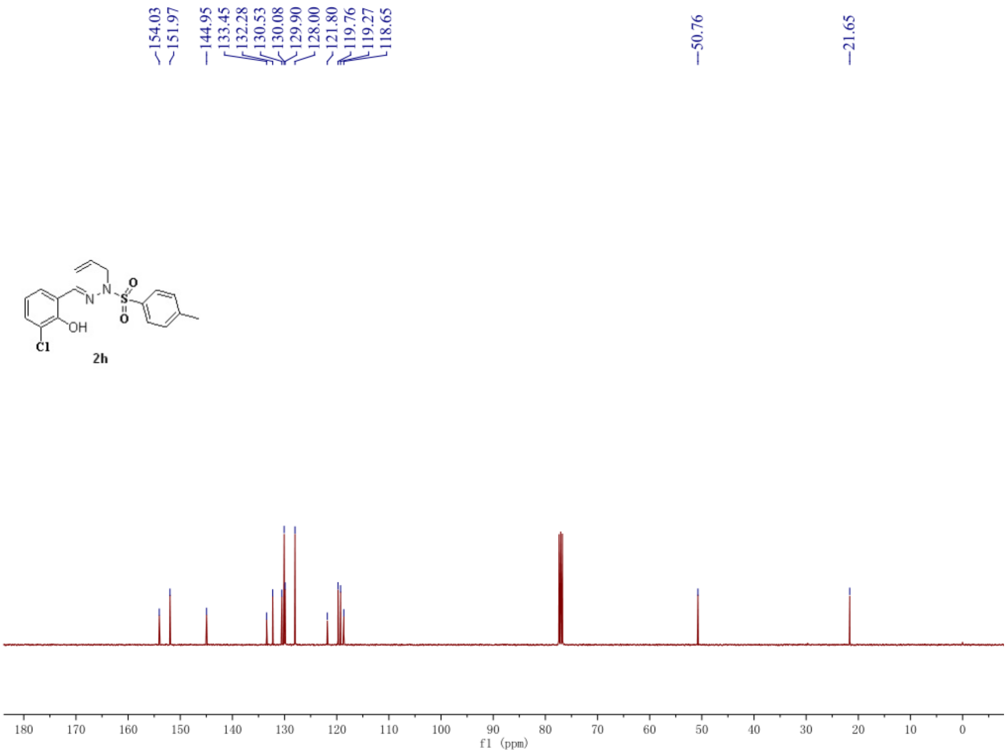

^{13}C NMR spectrum of compound **2h** (100 MHz, $CDCl_3$)

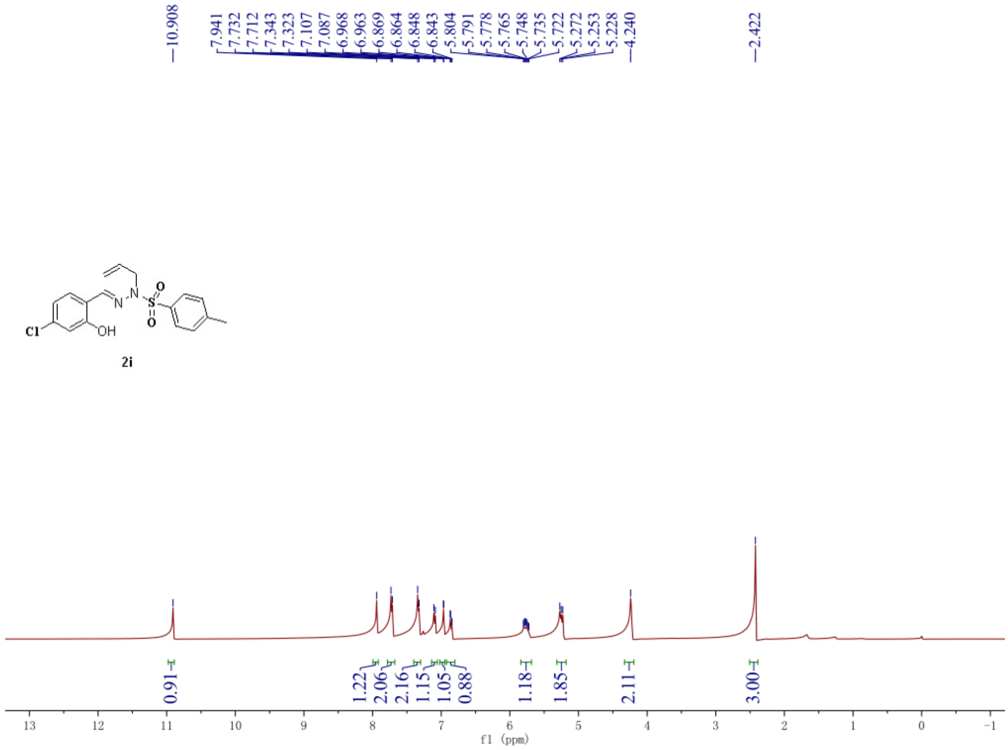

^{1}H NMR spectrum of compound **2i** (400 MHz, $CDCl_3$)

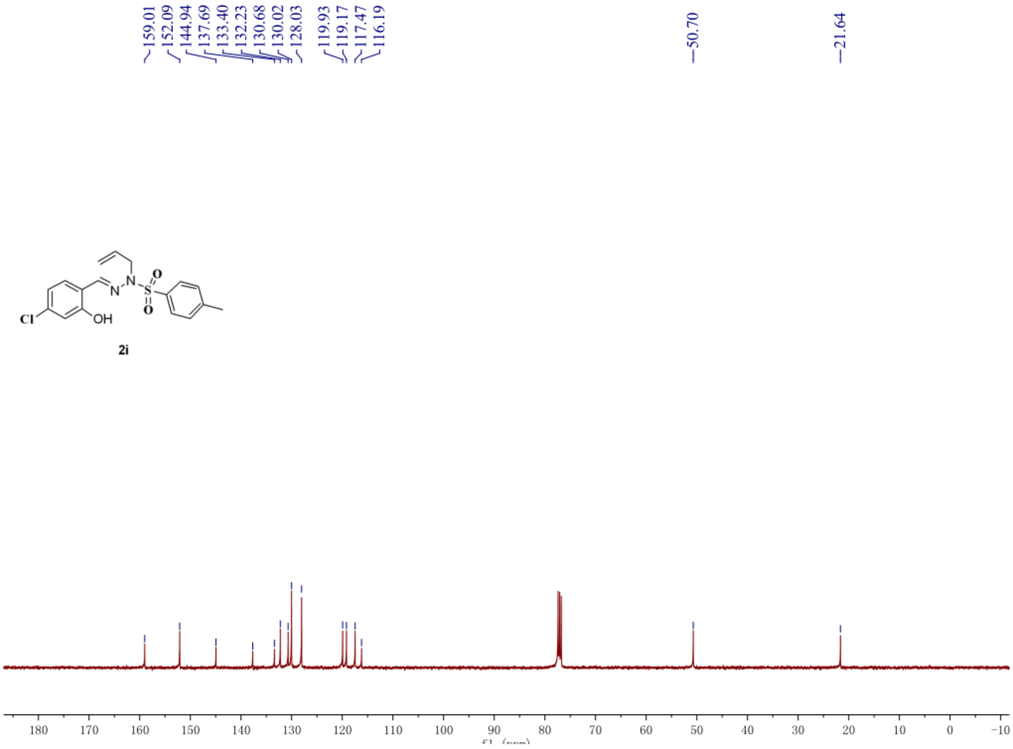

^{13}C NMR spectrum of compound **2i** (100 MHz, $CDCl_3$)

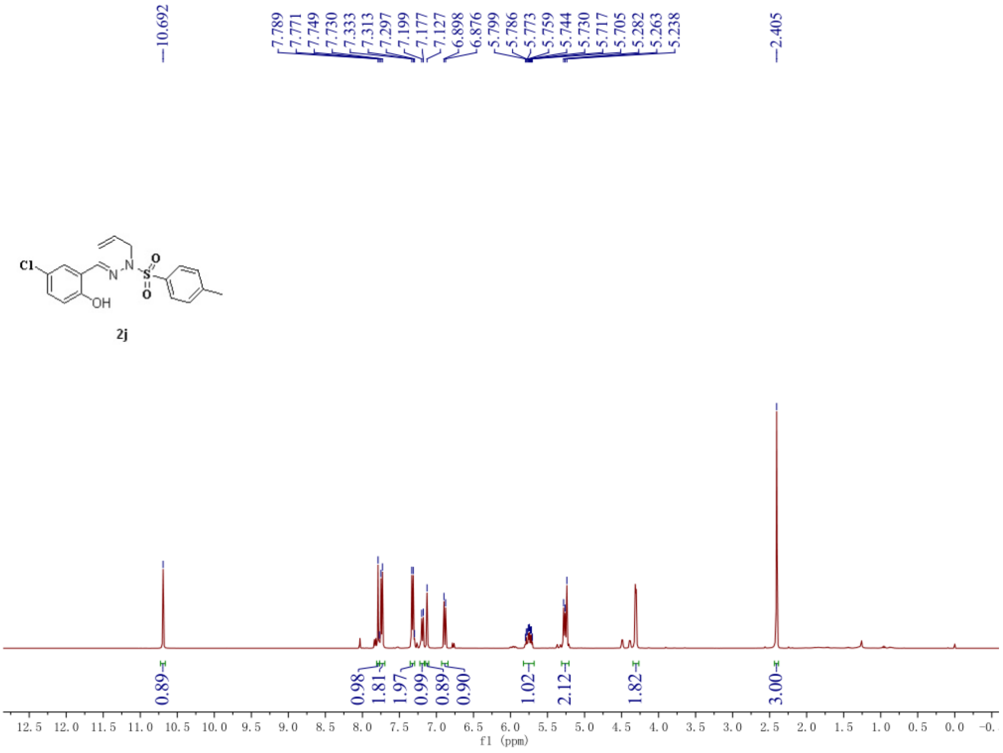

^{1}H NMR spectrum of compound **2j** (400 MHz, $CDCl_3$)

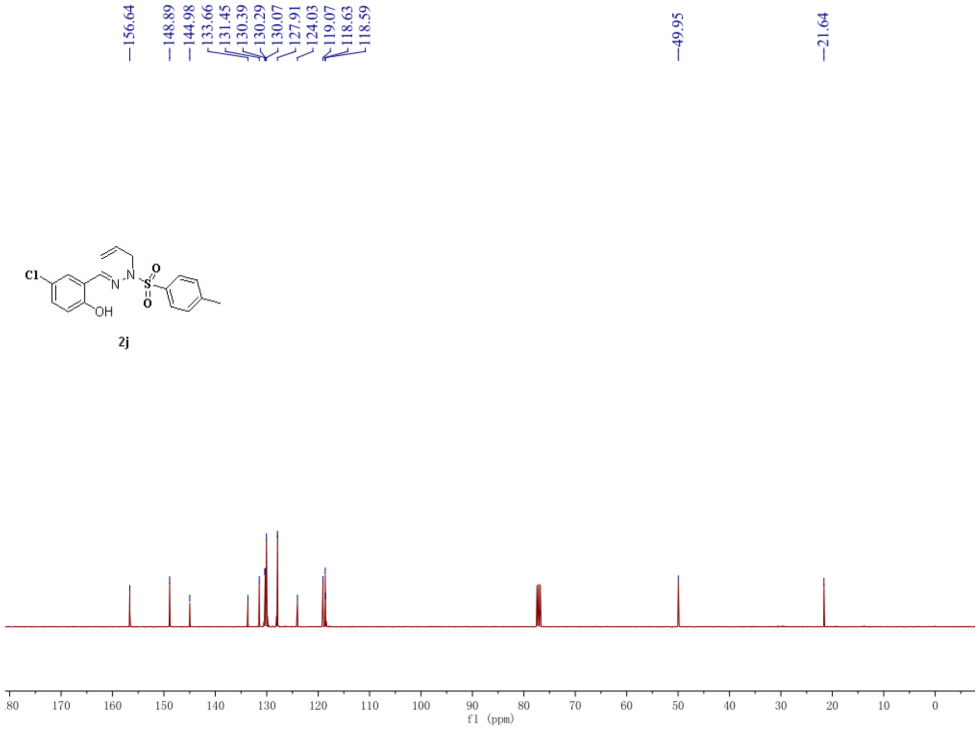

^{13}C NMR spectrum of compound **2j** (100 MHz, $CDCl_3$)

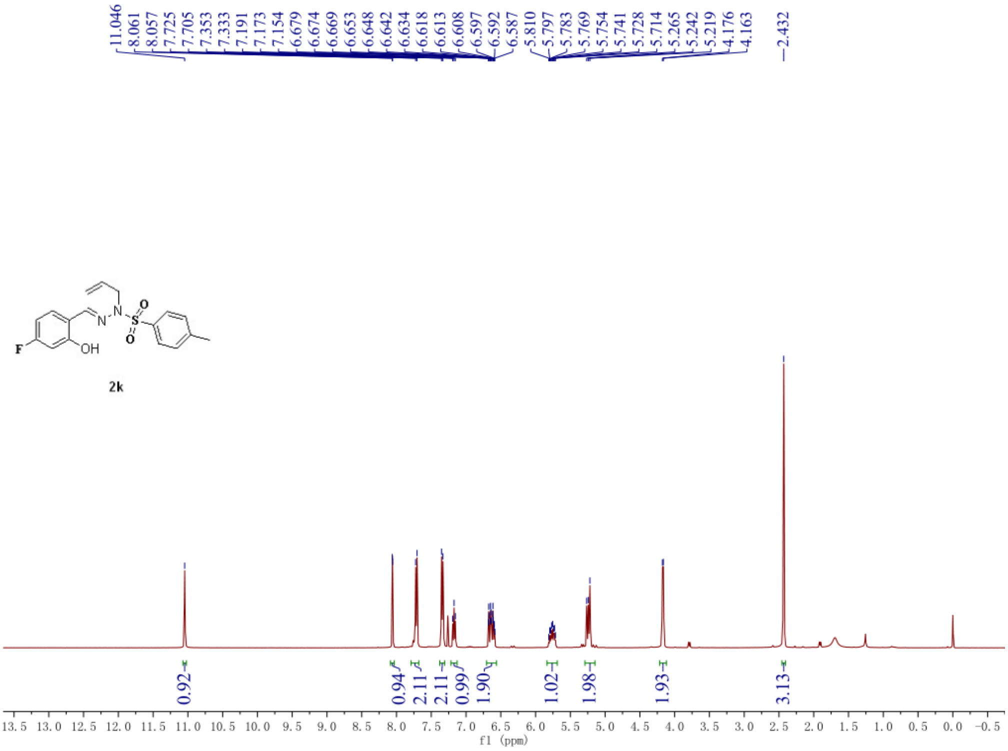

^{1}H NMR spectrum of compound **2k** (400 MHz, $CDCl_3$)

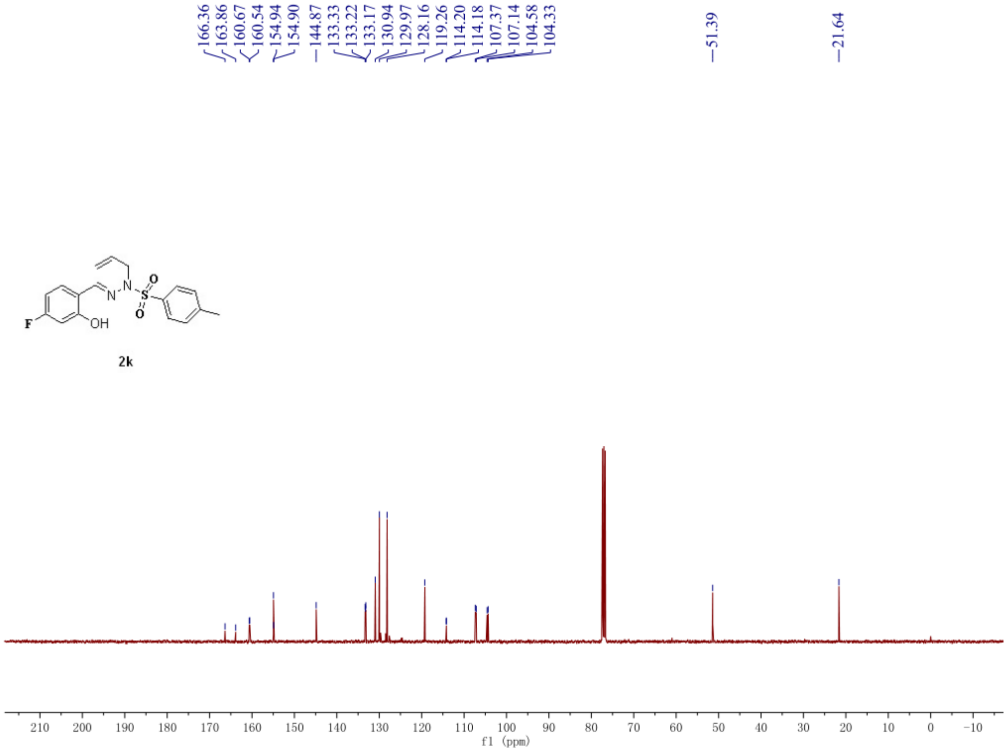

^{13}C NMR spectrum of compound **2k** (100 MHz, $CDCl_3$)

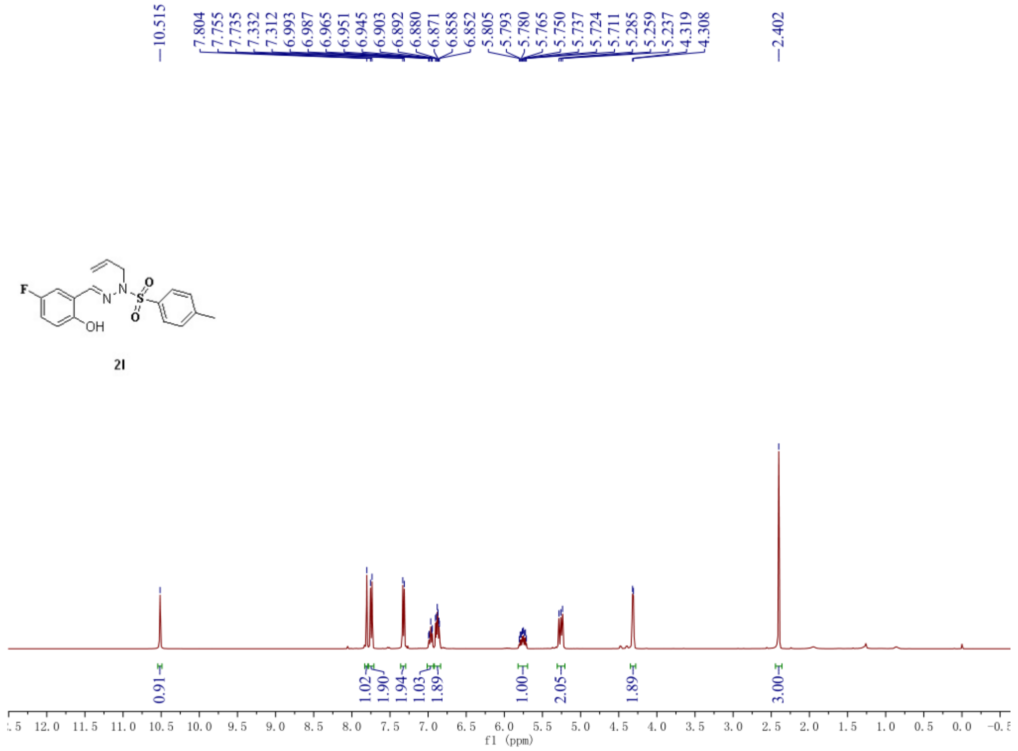

^{1}H NMR spectrum of compound **2l** (400 MHz, $CDCl_3$)

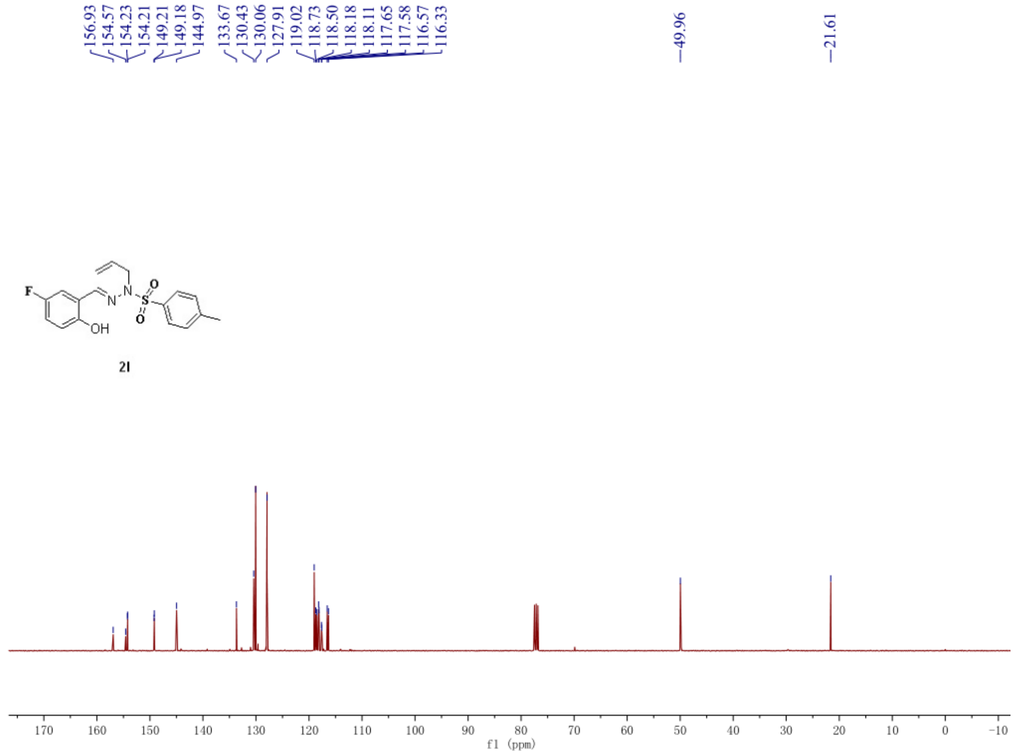

^{13}C NMR spectrum of compound **2l** (100 MHz, $CDCl_3$)

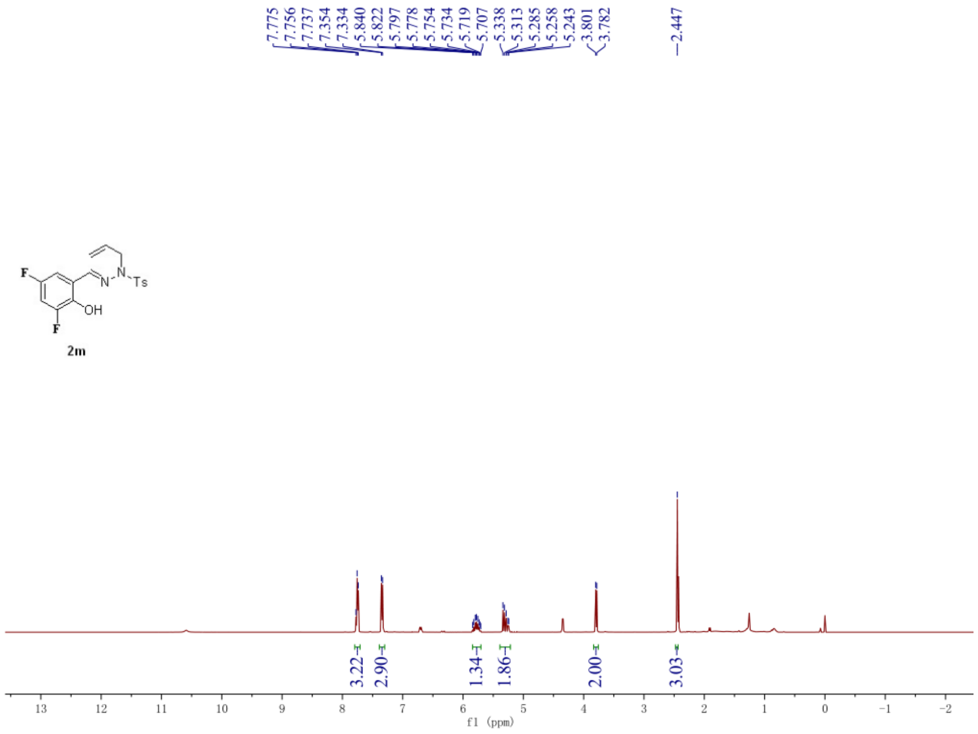

^{1}H NMR spectrum of compound **2m** (400 MHz, $CDCl_3$)

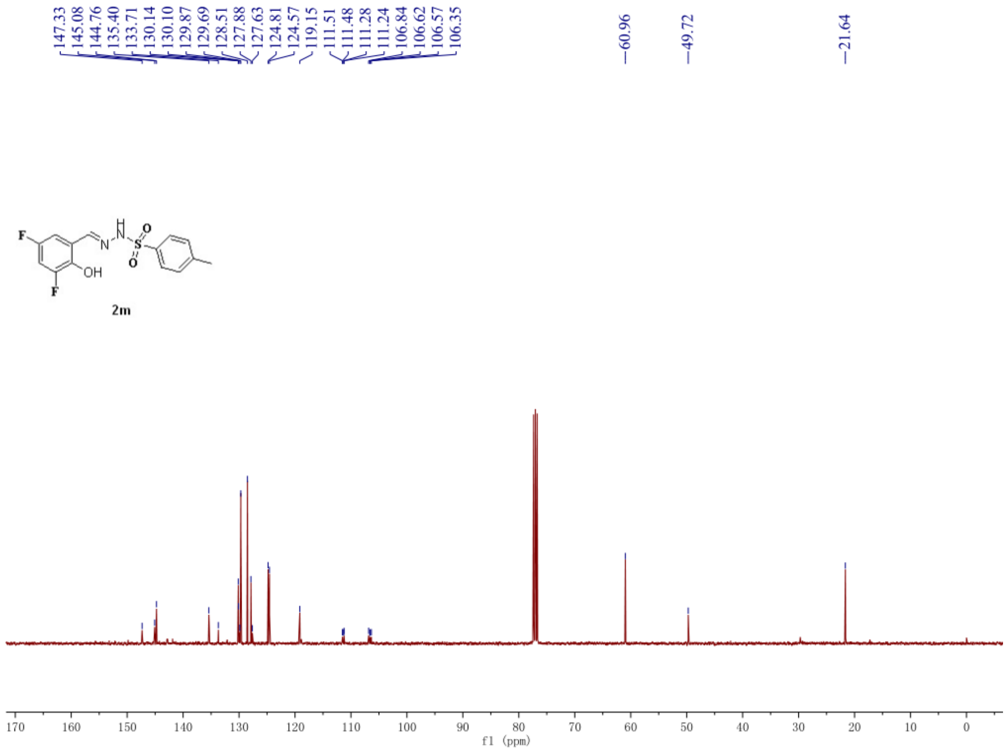

^{13}C NMR spectrum of compound **2m** (100 MHz, $CDCl_3$)

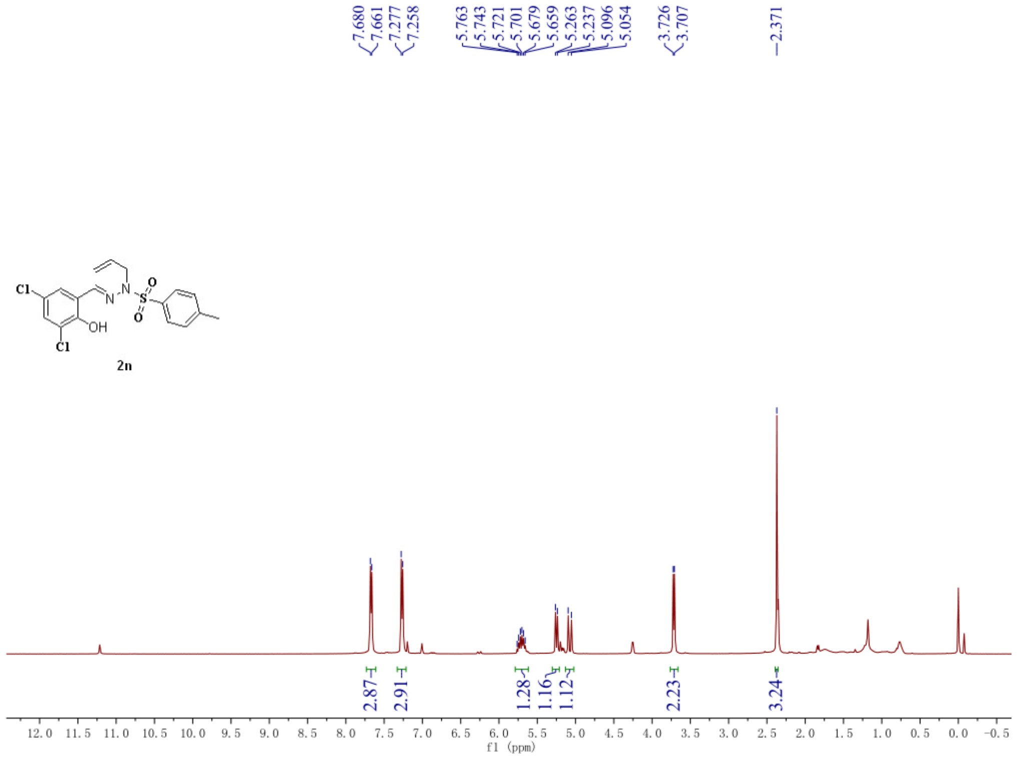

^{1}H NMR spectrum of compound **2n** (400 MHz, $CDCl_3$)

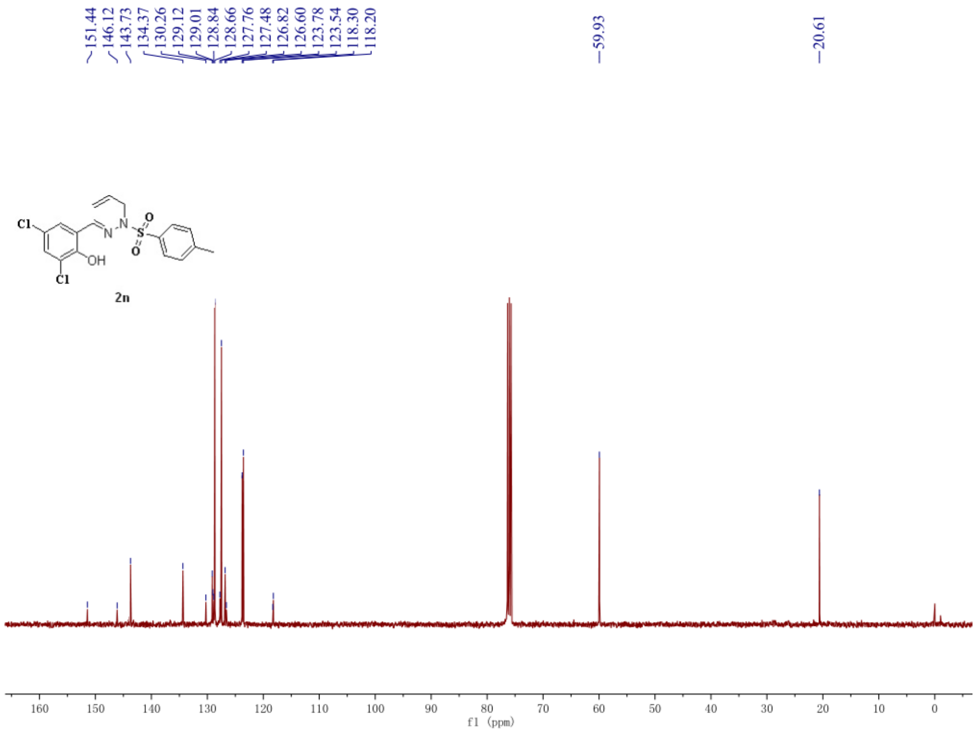

^{13}C NMR spectrum of compound **2n** (100 MHz, $CDCl_3$)

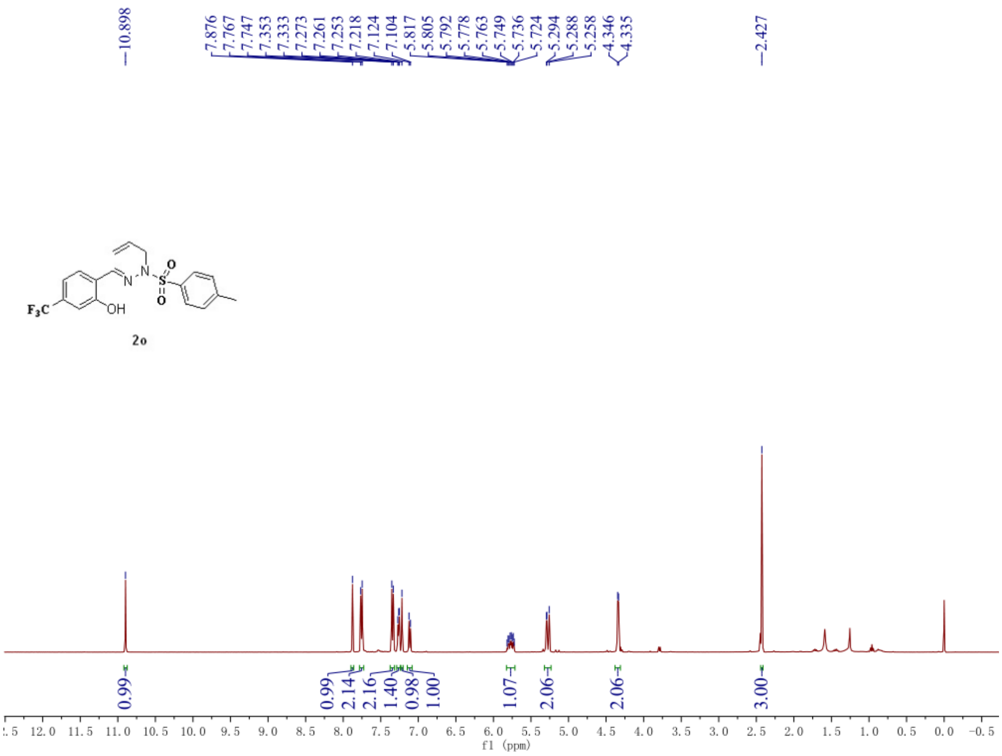

^{1}H NMR spectrum of compound **2o** (400 MHz, $CDCl_3$)

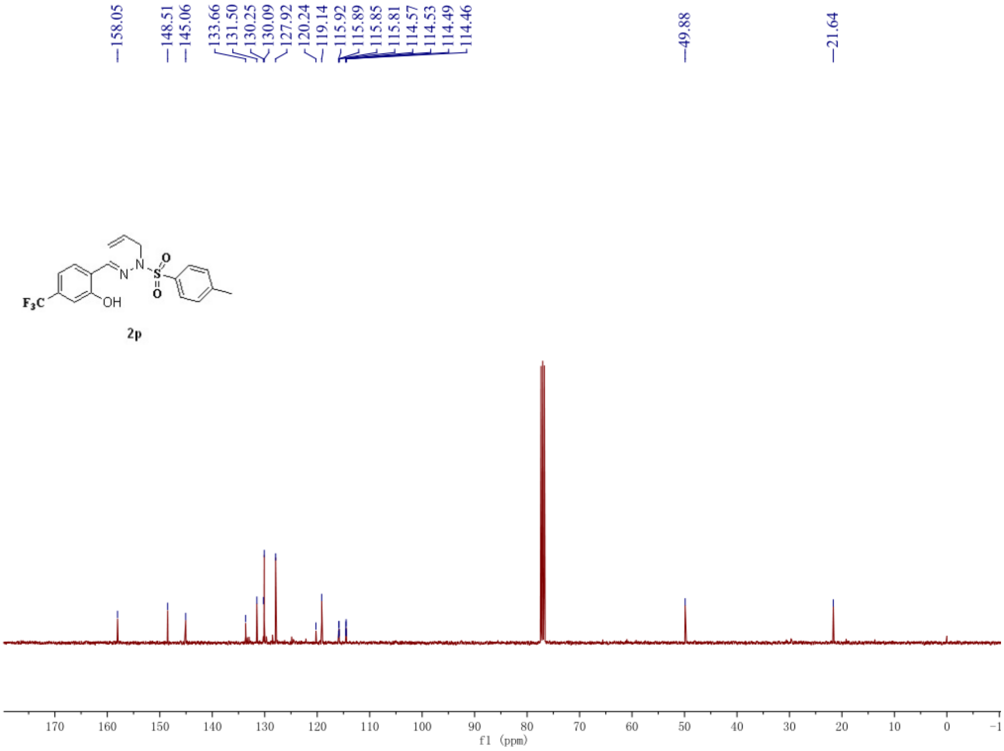

^{13}C NMR spectrum of compound **2o** (100 MHz, $CDCl_3$)

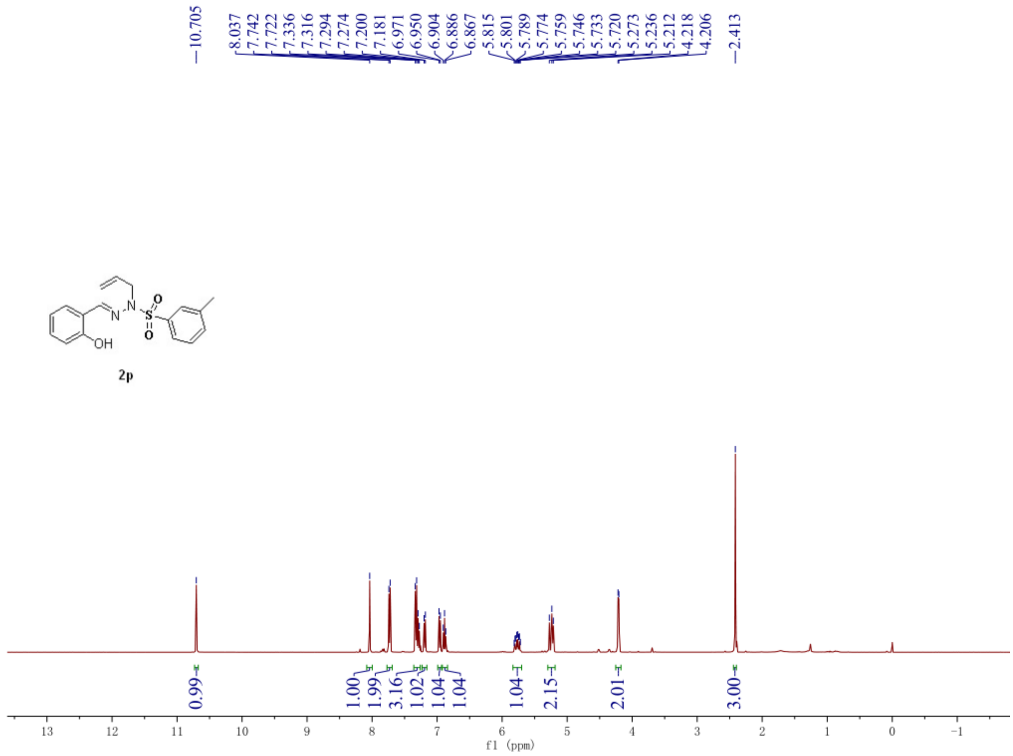

^{1}H NMR spectrum of compound **2p** (400 MHz, $CDCl_3$)

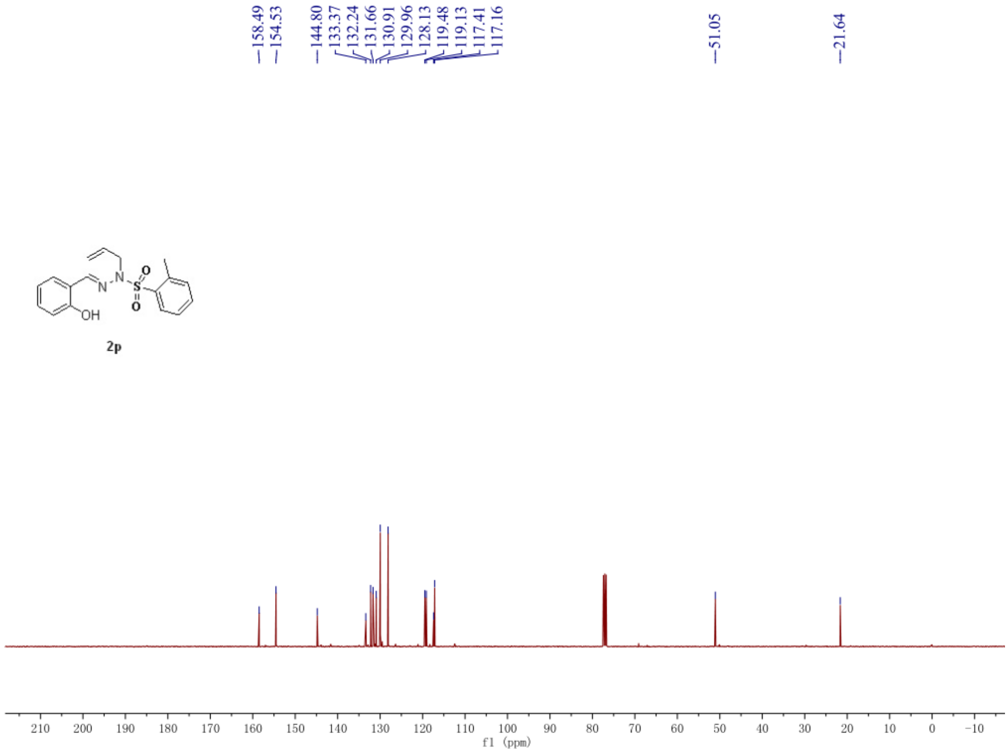

^{13}C NMR spectrum of compound **2p** (100 MHz, $CDCl_3$)

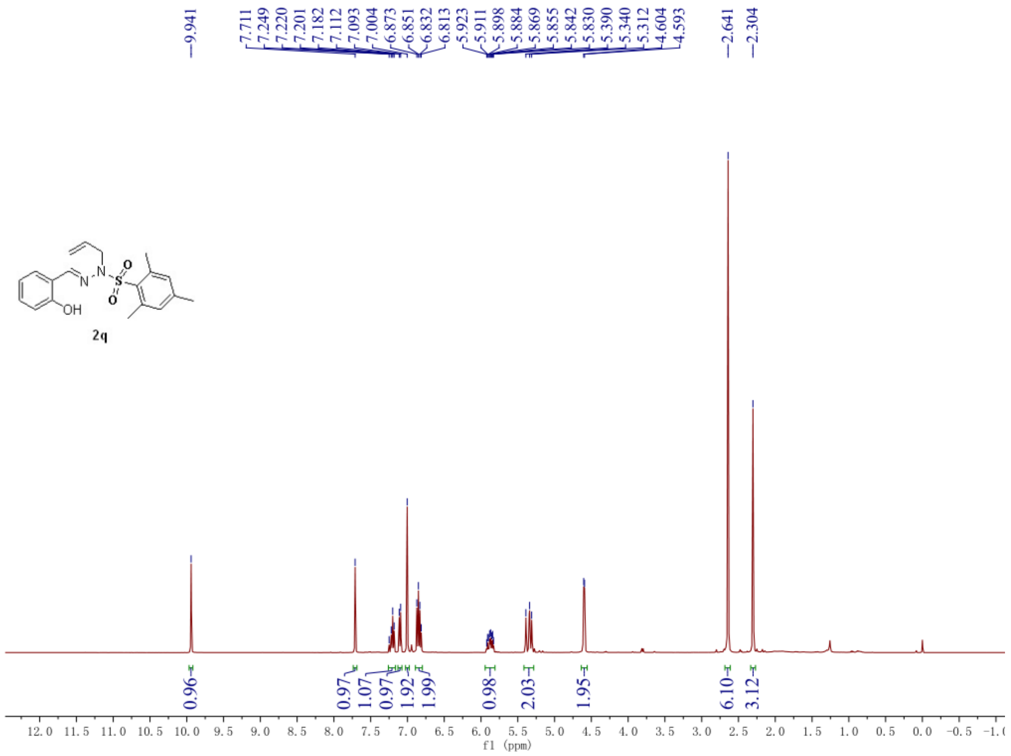

^{1}H NMR spectrum of compound **2q** (400 MHz, $CDCl_3$)

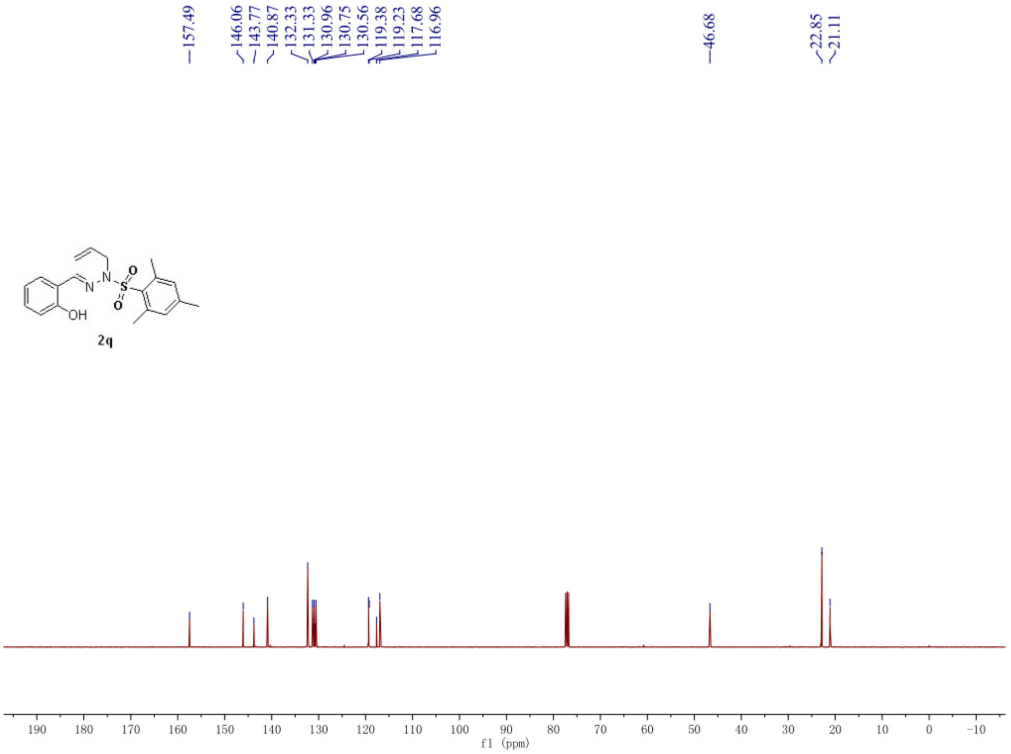

^{13}C NMR spectrum of compound **2q** (100 MHz, $CDCl_3$)

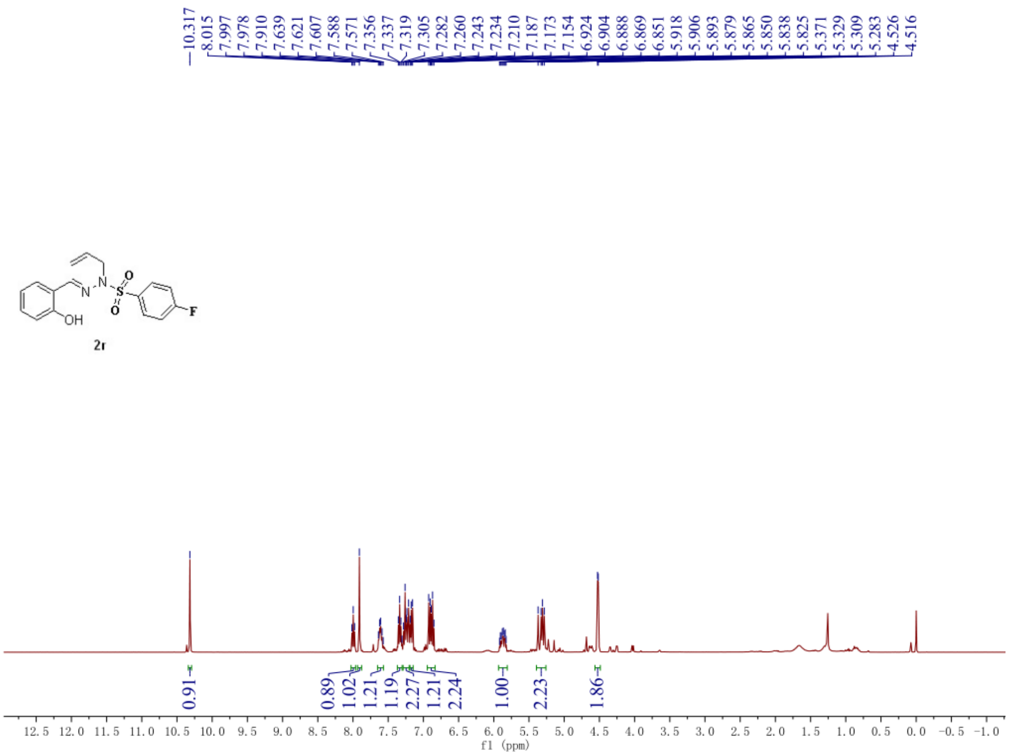

^{1}H NMR spectrum of compound **2r** (400 MHz, $CDCl_3$)

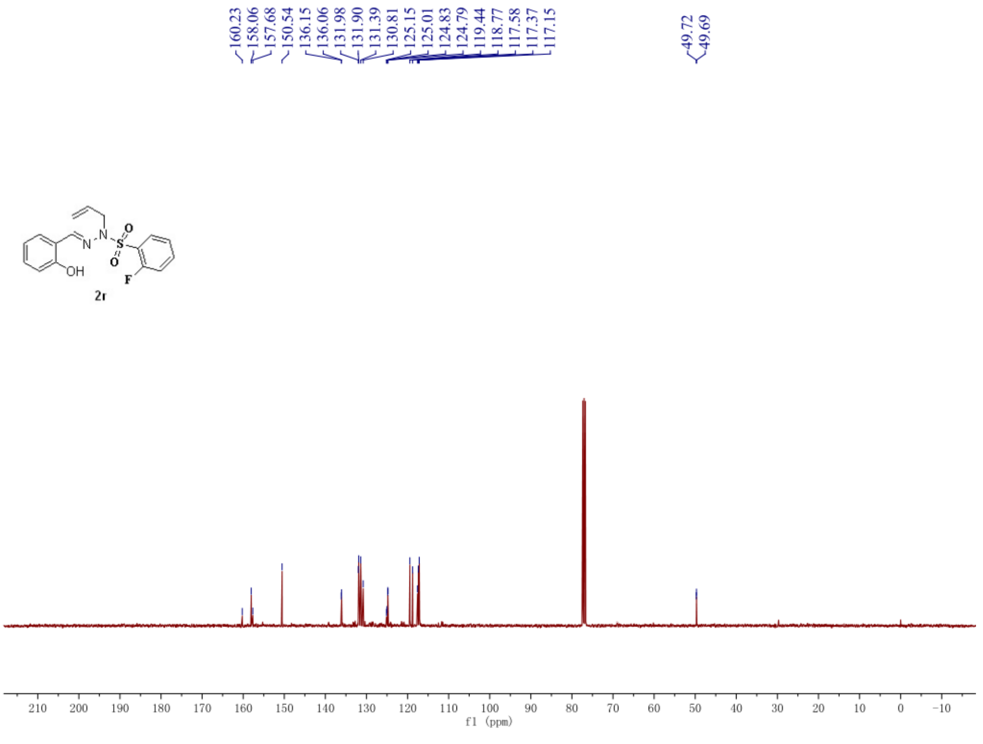

^{13}C NMR spectrum of compound **2r** (100 MHz, $CDCl_3$)

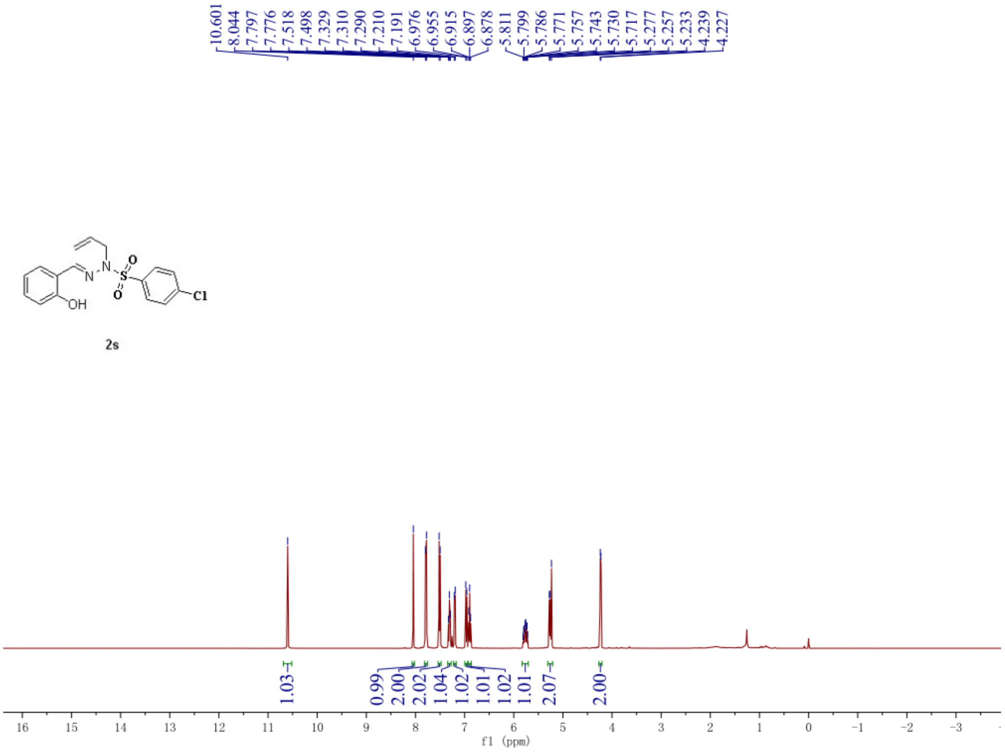

^{1}H NMR spectrum of compound **2s** (400 MHz, $CDCl_3$)

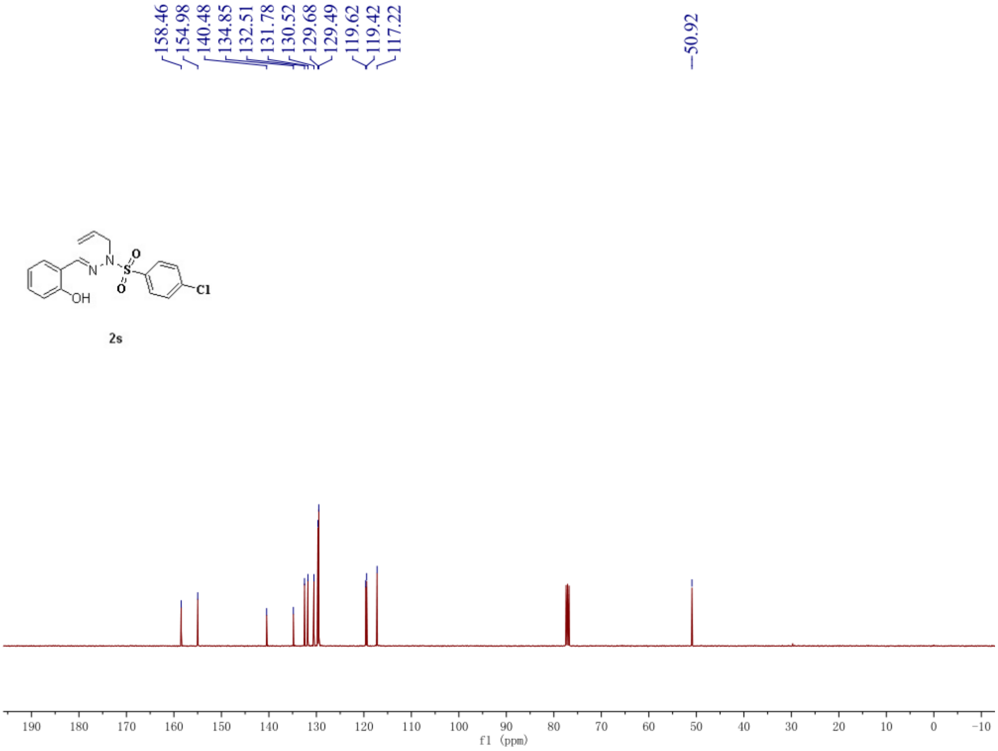

^{13}C NMR spectrum of compound **2s** (100 MHz, $CDCl_3$)

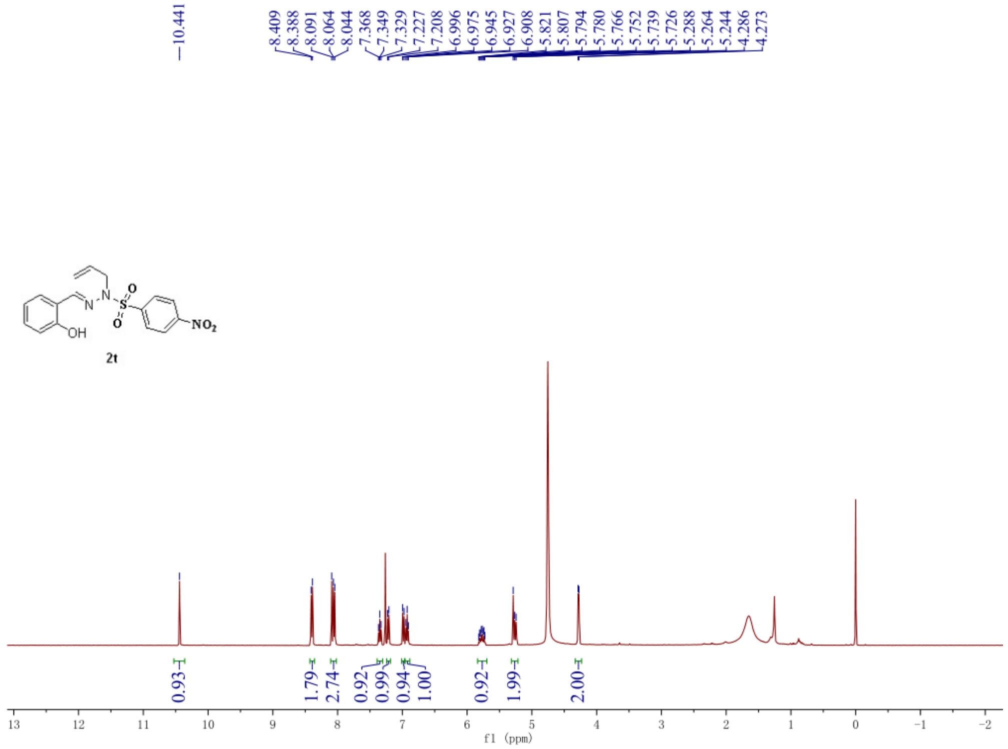

^{1}H NMR spectrum of compound **2t** (400 MHz, $CDCl_3$)

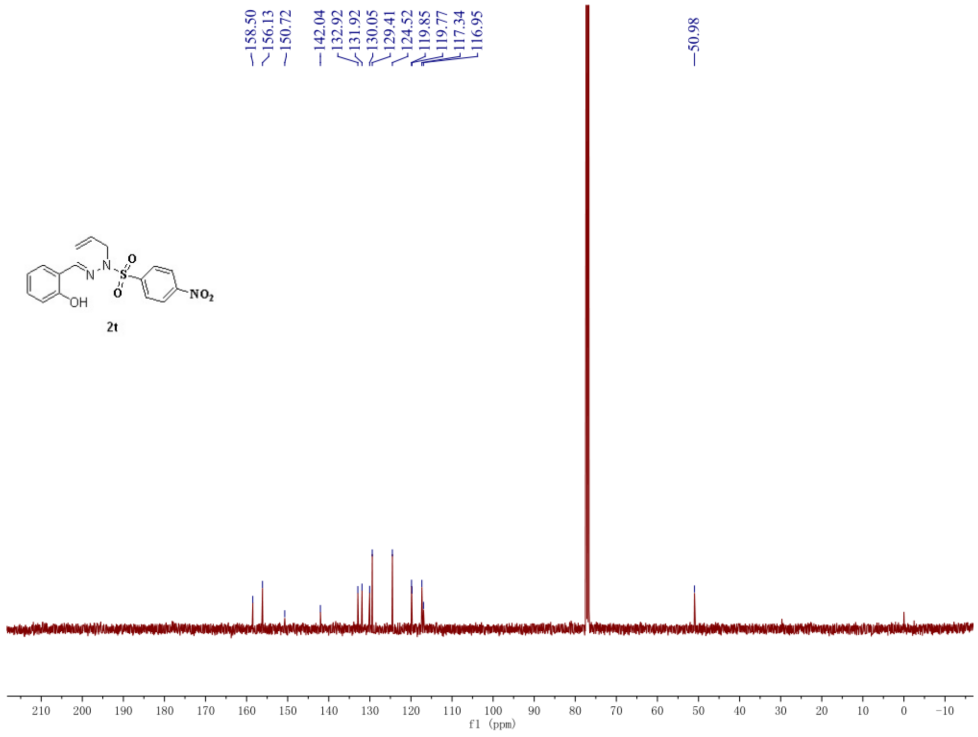

^{13}C NMR spectrum of compound **2t** (100 MHz, $CDCl_3$)

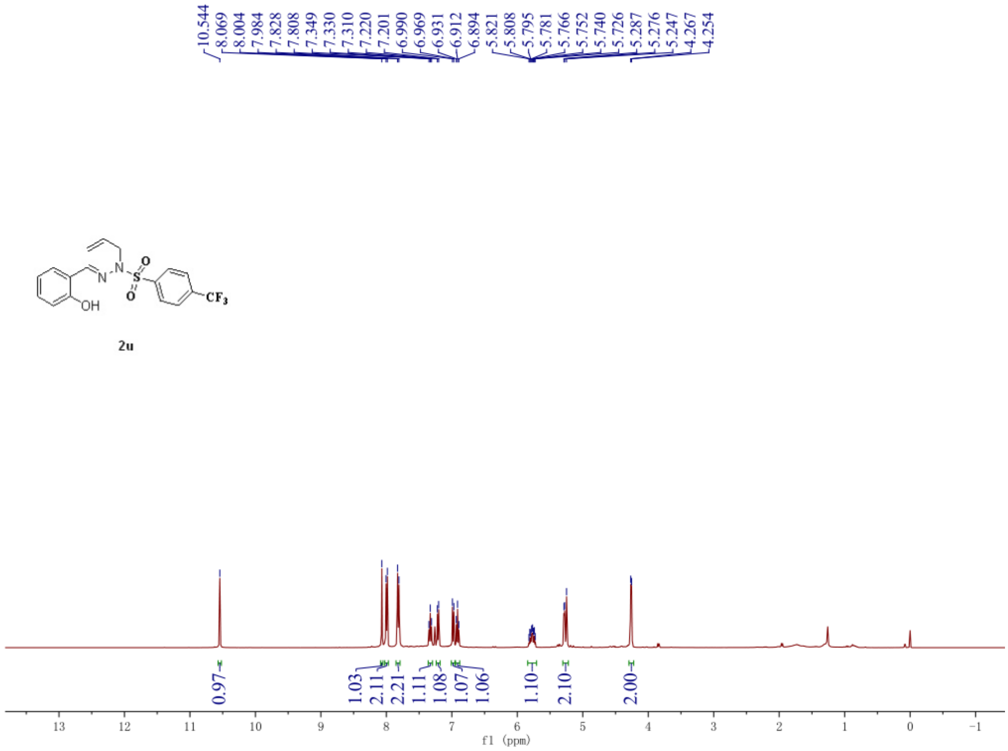

^{1}H NMR spectrum of compound **2u** (400 MHz, $CDCl_3$)

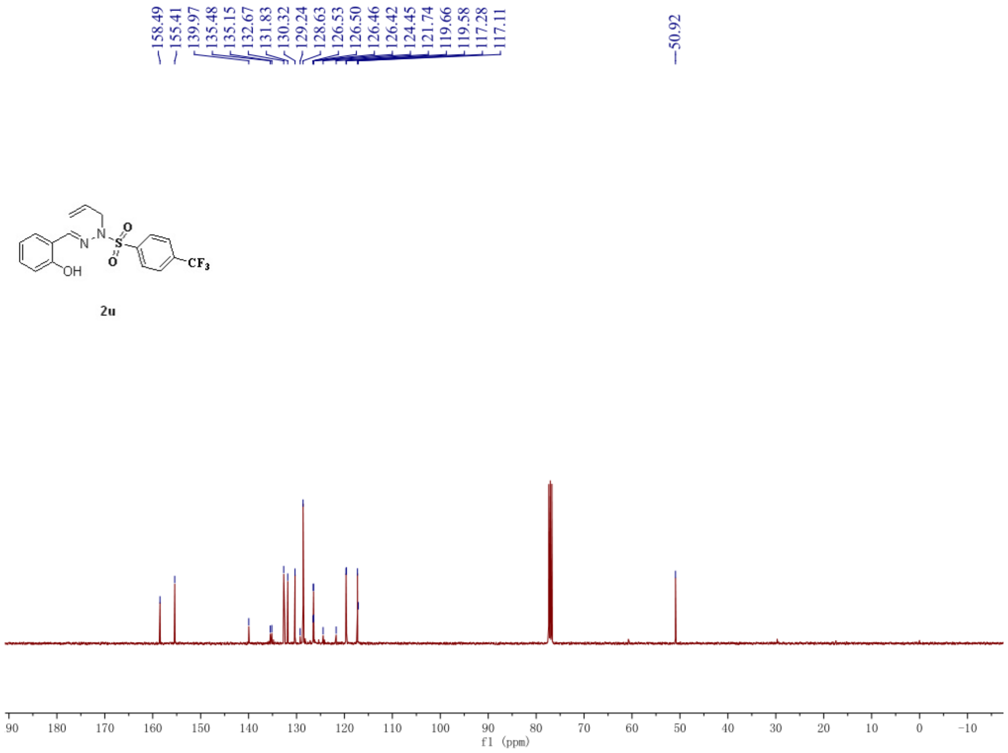

^{13}C NMR spectrum of compound **2u** (100 MHz, $CDCl_3$)

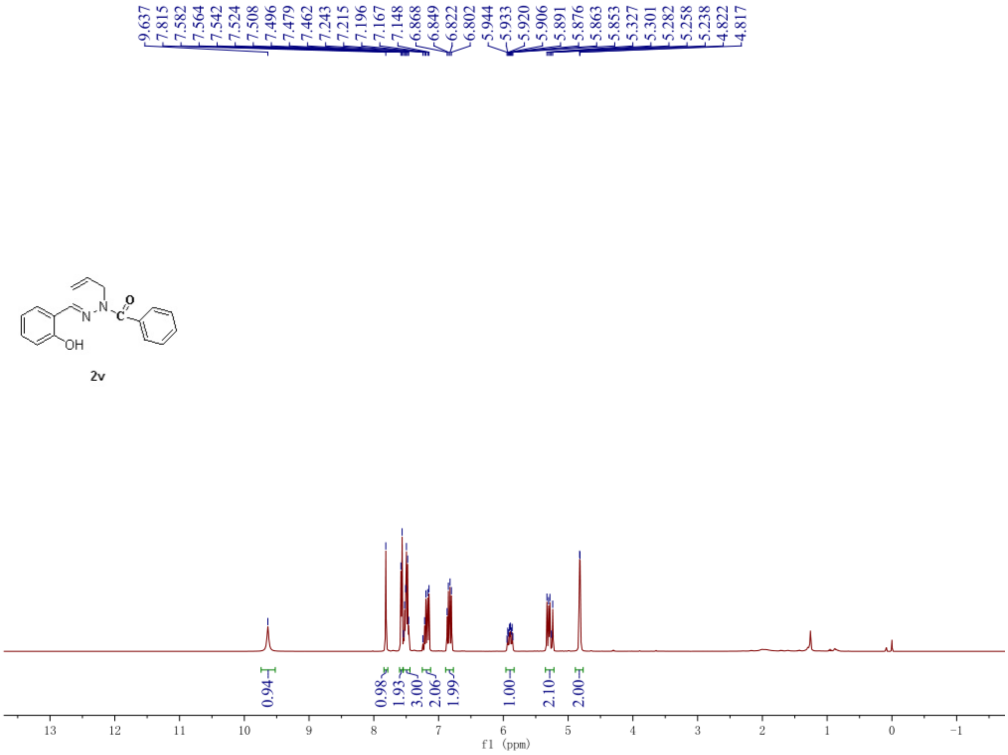

^{1}H NMR spectrum of compound **2v** (400 MHz, $CDCl_3$)

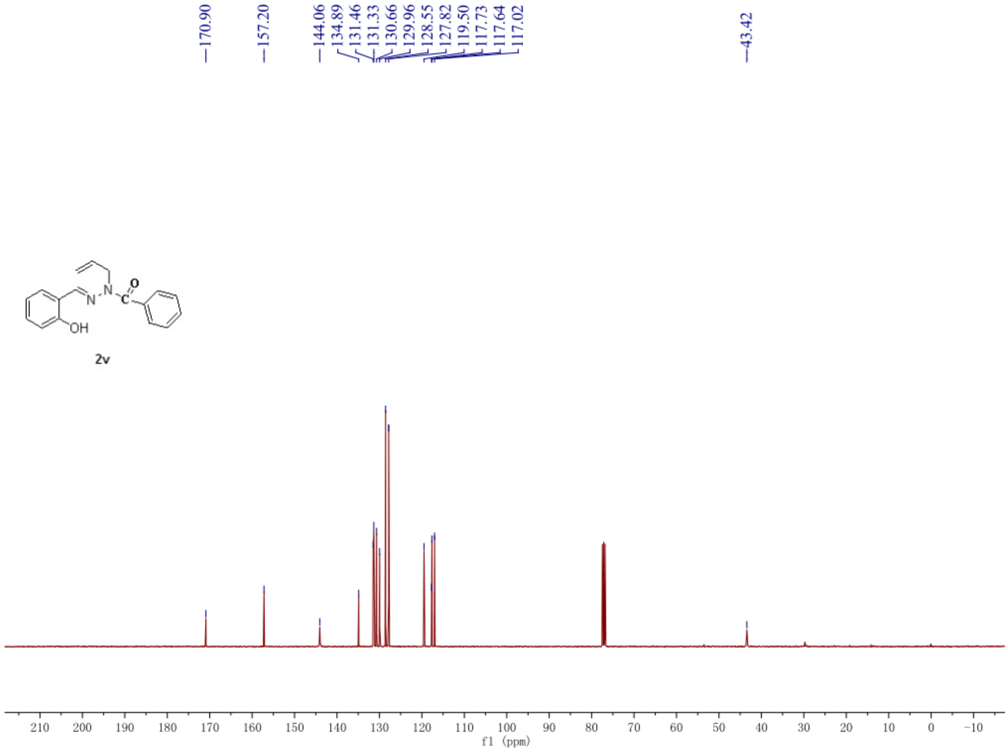

^{13}C NMR spectrum of compound **2v** (100 MHz, $CDCl_3$)

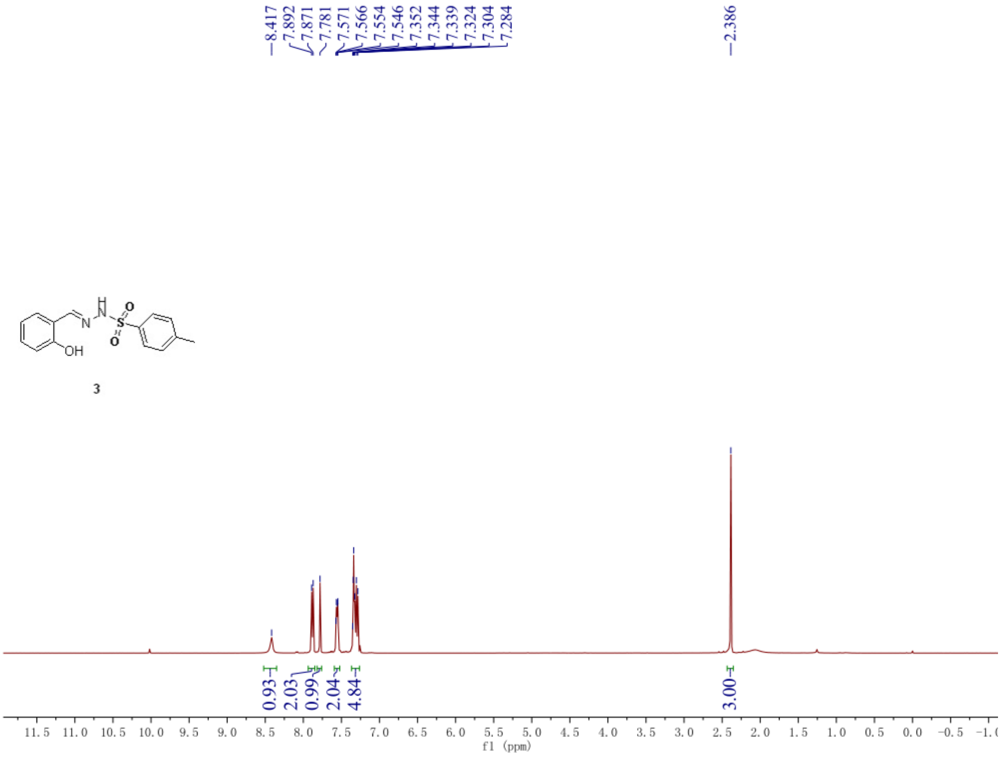

^{1}H NMR spectrum of compound **3** (400 MHz, $CDCl_3$)

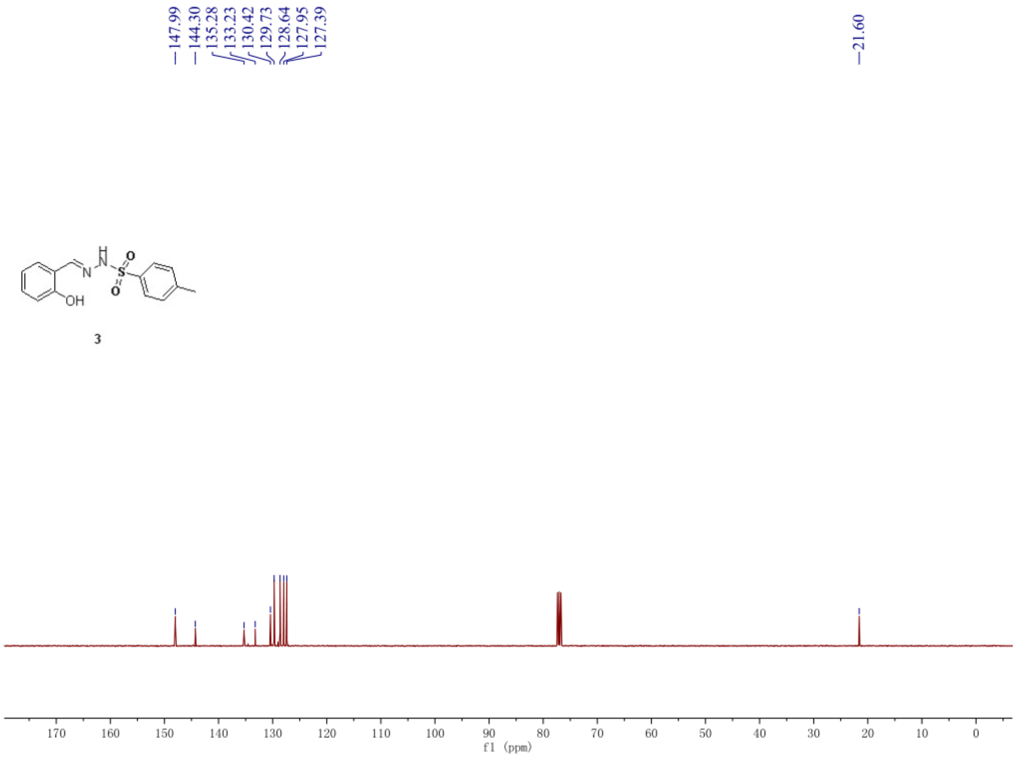

^{13}C NMR spectrum of compound **3** (100 MHz, $CDCl_3$)

Ⅳ. X-Ray Crystallographic Data of 2a

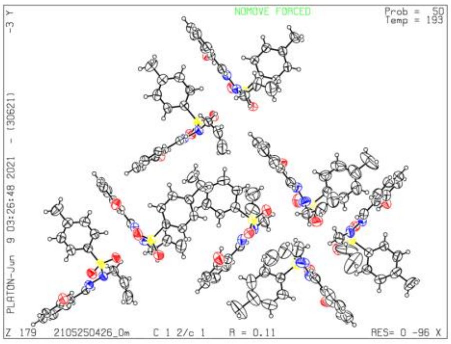

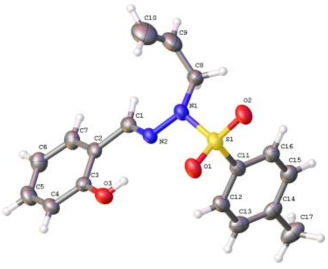

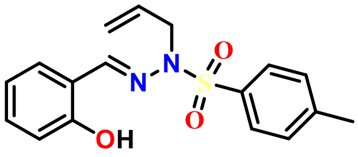

2a

Crystal structure and data of *(E)*-*N*-allyl-*N′*-(2-hydroxybenzylidene)-4-methylbenzene sulfonohydrazide (**2a**) (CCDC 2110634). The crystal was grown from DCM and EtOH. 15 mg of **2a** was dissolved in DCM and EtOH (1/20, 12 mL) and the solvent was evaporated slowly in a room atmosphere. A suitable crystal was selected, mounted on a glass fiber with aramid oil, and placed on a Bruker D8 risk diffractometer for testing (flowing metal Ga target).

Table 1. Crystal data and structure refinement for **2a**.

Identification code	2105250426_0m	
Empirical formula	$C_{17}H_{18}N_2O_3S$	
Formula weight	330.39	
Temperature	193.01 K	
Wavelength	1.34139 Å	
Crystal system	Monoclinic	
Space group	C 1 2/c 1	
Unit cell dimensions	a = 76.908(2) Å	α= 90°.
	b = 7.1310(2) Å	β= 122.5090(10)°.
	c = 57.8427(14) Å	γ = 90°.
Volume	26752.1(13) $Å^3$	
Z	64	
Density (calculated)	1.313 Mg/m^3	
Absorption coefficient	1.218 mm^{-1}	
F(000)	11136	
Crystal size	0.05 x 0.01 x 0.01 mm^3	
Theta range for data collection	3.018 to 55.036°.	
Index ranges	-93<=h<=93, -7<=k<=8, -70<=l<=70	
Reflections collected	78291	
Independent reflections	25241 [R(int) = 0.0683]	
Completeness to theta = 53.594°	99.0 %	
Absorption correction	Semi-empirical from equivalents	
Max. and min. transmission	0.7508 and 0.4653	

Refinement method	Full-matrix least-squares on F^2
Data / restraints / parameters	25241 / 32 / 1673
Goodness-of-fit on F^2	1.017
Final R indices [I>2sigma(I)]	R1 = 0.1088, wR2 = 0.2878
R indices (all data)	R1 = 0.1767, wR2 = 0.3423
Extinction coefficient	n/a
Largest diff. peak and hole	0.901 and -0.547 e.Å^{-3}
